# Modulation of Mixed-Cation Lead Halide Crystal Structures and Properties by Changing Small Size Cation

**DOI:** 10.1021/acs.inorgchem.6c04471

**Published:** 2026-10-28

**Authors:** Mirosław Mączka, Dawid Drozdowski, Andrzej Nowok, Dagmara Stefańska, Barbara Popanda, Adam Sieradzki

**Affiliations:** † Institute of Low Temperature and Structure Research, 215275 Polish Academy of Sciences, ul. Okólna 2, Wrocław 50-422, Poland; ‡ Department of Experimental Physics, 49567 Wrocław University of Science and Technology, Wybrzeże Wyspiańskiego 27, Wrocław 50-370, Poland

## Abstract

Mixing of organic cations is a promising strategy for extending the diversity of hybrid lead halides and tuning their properties. Most famous examples of this approach are multilayered Ruddlesden–Popper and Dion-Jacobson perovskites and three-dimensional (3D) MPDA_2_APb_4_Br_13_ perovskitoids (MPDA = *N*-methylpropanediammonium, A = methylammonium, formamidinium, ethylammonium, and dimethylammonium). We show that the 3D perovskitoid structure is no longer stable when imidazolium (IM^+^) and guanidinium (GA^+^) are used, but instead two- and one-dimensional structures are formed, respectively, and the stoichiometry changes to MPDA_2_IM_3_Pb_4_Br_15_ and MPDA_6_GA_6_Pb_7_Br_30_Br_2._ Most interestingly, MPDA_2_IM_3_Pb_4_Br_15_ comprises unprecedented layers composed of the corner-sharing PbBr_6_ octahedra organized into tetrameric units with IM^+^ located in the perovskite cages. It undergoes a transition from the high-temperature *P*2_1_/*c* phase to the low-temperature *P*2_1_/*n* phase associated with the dielectric anomaly. The MPDA_6_GA_6_Pb_7_Br_30_Br_2_ structure is composed of corner-sharing PbBr_6_ octahedra arranged into chains, and its crystal structure remains trigonal, *P*-3*C*1, in the entire temperature range studied. Optical studies show that different structural motifs strongly affect band gaps and emission properties of these compounds. In particular, MPDA_6_GA_6_Pb_7_Br_30_Br_2_ exhibits very stable broadband greenish-yellow emission, while MPDA_2_IM_3_Pb_4_Br_15_ exhibits both narrowband emission (attributed to free excitons (FEs) and bound excitons (BEs)) and self-trapped (STE) broadband emission, a thermally activated population transfer from STE to FE + BE, and strong thermochromism.

## Graphical abstract

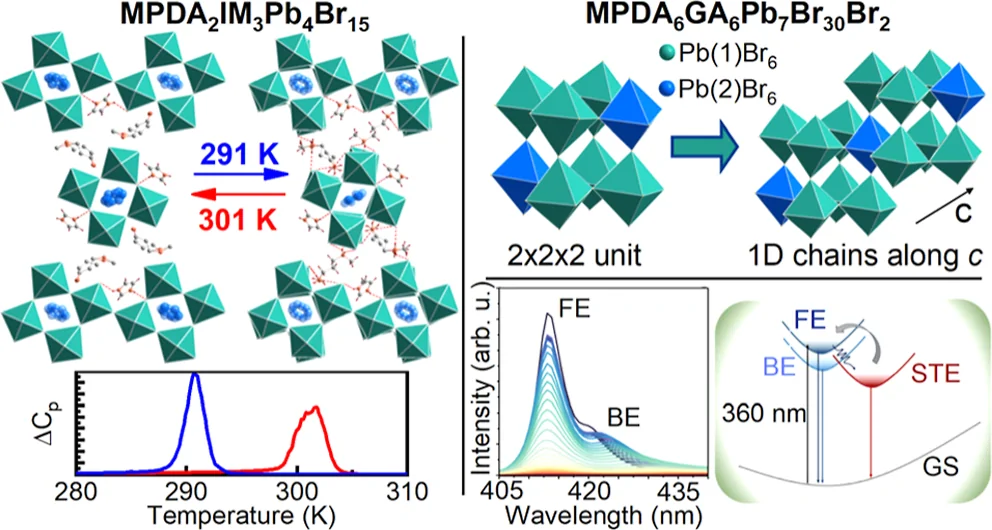

## Introduction

Hybrid organic–inorganic lead halides have been widely studied as promising semiconductors for photovoltaic and light-emitting applications.^1–5^ These compounds may crystallize in various stoichiometries, dimensionalities (three-, two-, one-, and zero-dimensional, 3D, 2D, 1D, and 0D), and connection modes of octahedral units (corner-, edge-, or face-sharing).^1–8^ Those built up of corner-sharing units, especially 3D and 2D, are called perovskites, while analogues with edge- or face-sharing octahedra are usually denoted as perovskitoids.^9^ The structural motifs strongly affect their properties. For instance, 3D lead iodide perovskites with the formula APbI_3_ (A denotes small methylammonium) (MA^+^), formamidinium (FA^+^), or aziridinium (AZR^+^) are famous for their photovoltaic properties and narrowband photoluminescence (PL) related to the recombination of free excitons (FEs),^1,10–12^ while 2D lead halide perovskites of general formula A′_2_PbX_4_ and A″PbX_4_ (A′ and A′ denote larger monovalent and divalent cation, respectively) may exhibit narrowband, broadband (related to self-trapped excitons, STEs), or both types of PL.^4,13–15^ The narrowband PL with high color purity is attractive for light-emitting diodes (LEDs), while the broad band PL, including white-light emission, is attractive for lighting.^5,14,16^ Some perovskitoids, for instance IMPbBr_3_ (IM = imidazolium),^8^ have also been reported to exhibit efficient broadband PL and nonlinear optical (NLO) properties. Recently, a family of 3D perovskitoids was also extended by using several diammonium cations. The general formula of such perovskitoids is APb_2_X_6_ (A denotes, for instance, 1,4-butanediammonium, 3-aminomethylpyridinium, 4-aminomethylpyridinium, (2-aminoethyl)­trimethylphosphanium, (2-aminoethyl)­trimethylammonium, *N*,*N*′-dimethyl-1,3-propanediammonium, *N*,*N*-dimethylethylenediammonium, and *N*-methylpropanediammonium (MPDA); X- = halide anion), and these compounds show improved charge transport compared with lower dimensional systems, favorable for various optoelectronic applications, e.g., photodetecton.^17–22^

Another way to increase the structural diversity of lead halide perovskites and perovskitoids is the employment of two or more organic cations. The most famous examples of the mixed-cation approach are multilayered Ruddlesden–Popper (RP) and Dion–Jacobson (DJ) perovskites with the general formula A′_2_A_*n*–1_Pb_*n*_X_3*n*+1_ and A″A_*n*–1_Pb_*n*_X_3*n*+1_, respectively, where *n* denotes the number of octahedral layers coupled together via apical halide ions separated by A′ or A″ cations. The inorganic layers possess cages similar to those present in 3D analogues, which are occupied by A^+^ cations.^4,15,23^ DJ and RP multilayered compounds have attracted a lot of attention since they are more stable than 3D analogues and their optical and photovoltaic properties can be significantly tuned by changing the *n* value and type of cage or interlayer cation.^4,14,23^ Furthermore, many of these compounds exhibit NLO and ferroelectric properties.^24–26^ Mixed-cation perovskitoids are much less explored. Nevertheless, it has been reported that such compounds can be formed using two monovalent organic cations, as evidenced by the formation of 3D (EA)­(MA)­Pb_2_I_6_ and (c-C3A)_3_(MA)_3_Pb_5_I_16_ (EA = ethylammonium, c-C3A = cyclopropylammonium), which have attracted attention as promising materials for solar-cell application.^27^ They may also be constructed using a pair of divalent and monovalent cations. An interesting example of this approach is 3D MPDA_2_FAPb_4_Br_13_, which possess small (large) voids occupied by FA^+^ (MPDA^2+^) cations. This compound has been shown to exhibit high two-photon absorption, enabling sensitive near-infrared photoresponse.^28^ Very recently, we have shown that the small cages can also be occupied by several other small cations, such as MA^+^, dimethylammonium (DMA^+^), and ethylammonium (EA^+^).^29^ This discovery showed that similarly as with 2D RP and DJ phases, relaxed tolerance factor implies also for 3D perovskitoids.^29^ MPDA_2_APb_4_Br_13_ compounds (A = MA, DMA, and EA) were shown to be attractive narrowband light-emitting compounds with strong thermochromism and the MA analogue showed good photodetection performance at a minimal external bias.^29^

Herein, we show that, although the ionic radius of IM^+^ (258 pm) is smaller than the ionic radii of EA^+^ (274 pm) and DMA (272 pm),^30^ the MPDA_2_APb_4_Br_13_ structure is not formed. Instead, the stoichiometry of the grown crystals is MPDA_2_IM_3_Pb_4_Br_15_, and the structure is layered, with part of the IM^+^ cations occupying perovskite cages and the remaining part of the IM^+^ cations and all MPDA^2+^ cations serving as spacer cations. The relatively large size of GA^+^ (278 pm)^30^ also does not support the MPDA_2_APb_4_Br_13_-type structure, leading to new MPDA_6_GA_6_Pb_7_Br_30_Br_2_ stoichiometry, and 1D structure. We show that different structural motifs strongly affect the band gap and PL properties of these compounds. We also report dielectric studies of MPDA_2_IM_3_Pb_4_Br_15_ and discuss the mechanism of the discovered phase transition.

## Experimental Section

### Crystal Growth

All reagents (PbBr_2_ 99%, HBr 48 wt % in H_2_O, *N*-methyl-1,3-diaminopropane 98%, imidazole, and guanidinium carbonate) used for the synthesis were commercially purchased from Sigma-Aldrich and used without further purification. In our first attempts, we tried to obtain analogues of previously reported MPDA_2_APb_4_Br_13_ perovskites with GA^+^ or IM^+^ located in the small perovskite voids using the same procedure as reported in ref 29 (MPDA/Pb/M^+^ molar ratio 1:5:4 or 1:4:6; due to much lower solubility of MPDA-based bromides compared to GA or IM analogues, the stoichiometric ratio of the reagents cannot be used since it leads to crystallization of MPDAPb_2_Br_6_). Unfortunately, this procedure resulted in the crystallization of MPDAPb_2_Br_6_ crystals (in the case of the GA analogue) or a mixture of MPDAPbBr_4_ and IMPbBr_3_ (in the case of the IM analogue). Therefore, we have modified the reaction mixture by keeping a constant content of PbBr_2_ (6 mmol) and changing the amount of the other reagents. After several attempts, successful synthesis of crystals containing both MPDA^2+^ and GA^+^ or IM^+^ cations was obtained, as described below. Namely, single crystals of MPDA_6_GA_6_Pb_7_Br_30_Br_2_ were grown from a reaction mixture containing 6 mmol of PbBr_2_, 1 mmol of *N*-methyl-1,3-diaminopropane, and 15 mmol of guanidinium carbonate dissolved in HBr. The reaction mixture was then kept at 40 °C for slow evaporation of the solvent, and colorless crystals with dimensions up to 20 mm that grew in the vial were separated from the mother liquid after 3 days. The same procedure was used for synthesis of MPDA_2_IM_3_Pb_4_Br_15_, but the solution mixture contained 6 mmol of PbBr_2_, 2 mmol of *N*-methyl-1,3-diaminopropane, and 5 mmol of imidazole dissolved in HBr. The crystals up to 6 mm long that grew in the vial were separated after 1 week. Images of the grown crystals are shown in Figure S1. The comparison of powder XRD patterns of the grown compounds with the calculated ones based on the single-crystal data indicates the absence of obvious additional crystalline phases (Figure S2).

### Powder X-ray Diffraction

Powder X-ray diffraction (PXRD) patterns of the ground crystals were measured in the reflection modes using an X’Pert PRO X-ray diffraction system equipped with a PIXcel ultrafast line detector and Soller slits for Cu Kα_1_ radiation (λ = 1.54056 Å).

### Differential Scanning Calorimetry Measurements

DSC experiments were performed on powdered samples using a Mettler Toledo DSC-3 calorimeter with a resolution of 0.4 μW. Nitrogen was used as a purging gas, and the heating/cooling rate was 5 K/min. The mass of the measured samples was 15.56 mg and 23.4 mg for MPDA_2_IM_3_Pb_4_Br_15_ and MPDA_6_GA_6_Pb_7_Br_30_Br_2_, respectively. The excess heat capacity associated with the phase transition discovered in MPDA_2_IM_3_Pb_4_Br_15_ was calculated by subtraction from the data, with the baseline representing the system variation in the absence of phase transition.

### Single-Crystal X-ray Diffraction

Single-crystal X-ray diffraction data for MPDA_2_IM_3_Pb_4_Br_15_ were collected at 310 K, 270, and 100 K, on the Oxford Diffraction (OD) Xcalibur four-circle diffractometer with an Atlas CCD detector, using graphite-monochromated Mo Kα_1_ radiation (λ = 0.71073 Å). For MPDA_6_GA_6_Pb_7_Br_30_Br_2_, diffraction data was acquired at 295 K on the OD New Gemini diffractometer equipped with a dual microfocus source and an Atlas CCD detector, as well as with Mo Kα_1_ radiation. For both compounds, absorption effects were corrected via Gaussian integration over a multifaceted crystal model, with an empirical absorption correction based on spherical harmonics using the SCALE3 ABSPACK scaling algorithm. Data reduction and processing were performed with CrysAlis PRO 1.171.45.72a (Rigaku OD, 2026). Structure solution and refinement were carried out in Olex2 1.5, employing SHELXT and SHELXL, respectively.^31–33^ Hydrogen atoms were placed at geometrically calculated positions and refined as riding atoms. Key experimental and geometric parameters are summarized in Tables S1–S6.

### Broadband Dielectric Spectroscopy

The dielectric properties of MPDA_2_IM_3_Pb_4_Br_15_ were studied using a Novocontrol Alpha impedance analyzer coupled with a Novocontrol Quatro system and a nitrogen-gas cryostat. All measurements were performed on a polycrystalline sample in the form of a cylindrical pellet, characterized by a diameter of 5 mm and a thickness of 0.5 mm. Before these measurements, both flat surfaces of the pellet were silver pasted to ensure good electrical contact. Dielectric studies were carried out over the temperature range of 130–350 K during both cooling and heating cycles. Temperature regulation throughout the experiment was achieved using a controlled nitrogen-gas flow, and the spectra were collected every 2 K after at least 60 s of temperature stabilization. The frequency range was set from 10^1^ to 10^6^ Hz, while the temperature accuracy was selected to 0.2 K. The experimental data obtained were analyzed in the frequency domain using the WinFit software, where the dielectric spectra characterized by the relaxation process were parametrized with the Havriliak–Negami model. Such analysis was performed only for the complex dielectric permittivity, ε*­(*f*) = ε′(*f*) – iε″(*f*), with ε′ and ε″ denoting its real and imaginary parts, respectively. Accordingly, the experimental data were fitted using the following equation

1 $$\varepsilon^{*}\left(\omega \right)=\varepsilon_{\infty }+\frac{\Delta \varepsilon }{{\left(1+{\left(i\omega \tau_{HN}\right)}^{\alpha_{HN}}\right)}^{\beta_{HN}}}$$

where ε_∞_ is the high-frequency limit of the dielectric permittivity, Δε denotes the dielectric increment, ω is the angular frequency, τ_HN_ is the so-called Havriliak–Negami characteristic relaxation time, and the β_HN_ and α_HN_ describe the asymmetric and symmetric broadening of the relaxation process. The shape parameters β_HN_ and α_HN_ were further used to determine the relaxation times.

### Optical Absorption and PL Studies

Room-temperature (RT) diffuse reflectance spectra of the powdered materials were obtained with a Cary 5E UV–Vis–NIR spectrophotometer (Varian, Palo Alto, CA, USA). Temperature-dependent PL measurements were carried out using 360 nm excitation from a diode-laser source, and the emitted light was analyzed with a Hamamatsu PMA-12 multichannel detector equipped with a BT-CCD linear sensor (Hamamatsu Photonics, Iwata, Japan). The temperature of the samples during measurements was regulated with high accuracy using a Linkam THMS 600 Heating/Freezing Stage (Linkam Scientific Instruments, Tadworth, UK).

## Results and Discussion

### DSC

The DSC measurements show a lack of any heat anomalies for MPDA_6_GA_6_Pb_7_Br_30_Br_2_ in the 120–400 K range, while MPDA_2_IM_3_Pb_4_Br_15_ exhibits one heat anomaly at *T*_1_ = 301 K (291 K) during heating (cooling), respectively (Figure S3). Large thermal hysteresis and step-like change of the phase transition entropy and enthalpy indicate the first-order character of this transition (Figure 1). The associated changes in enthalpy Δ*H* and entropy Δ*S* were estimated to be ∼4.34 KJ mol^–1^ and ∼14.9 J mol^–1^ K^–1^ (average values), respectively. Very large value of Δ*S* suggests a predominantly order–disorder phase transition with a complex mechanism. To gain further insight, we performed X-ray diffraction studies.

**Figure 1:**
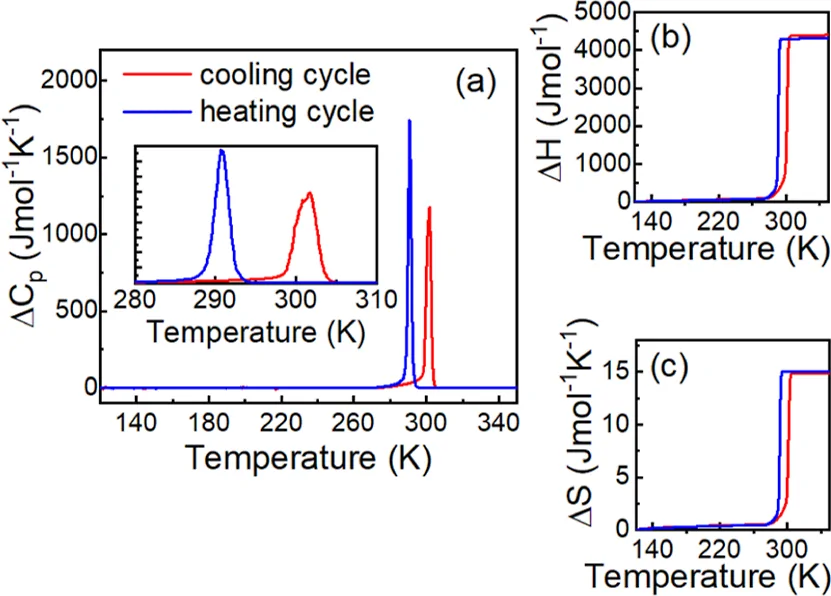
(a) Excess heat capacity, (b) enthalpy, and (c) entropy of MPDA_2_IM_3_Pb_4_Br_15_ in the heating (red) and cooling (blue) run.

### Single-Crystal X-ray Diffraction

MPDA_2_IM_3_Pb_4_Br_15_ adopts a 2D structure with [Pb_4_Br_15_]^7–^ layers propagating within the *ab* plane and separated along the *c* direction by the MPDA^2+^ and IM^+^ cations (Figure 2). Within each layer, the octahedra are organized into tetrameric units composed of four corner-sharing PbBr_6_ octahedra, forming a perovskite void. The tetramers are oriented with the void diagonal rotated by ca. 45° relative to the unit cell axes, giving each unit a diamond-shaped cross-section when viewed along the *bc* plane. Adjacent tetramers are connected through corner-sharing, bridging bromides in the [010] direction. High-temperature (HT) phase **I** is described in the monoclinic *P*2_1_/*c* space group. The asymmetric unit (Figure S4a) comprises two Pb^2+^ cations and seven Br^–^ anions, with bridging bromides occupying a 1= site symmetry and all remaining atoms residing on general positions. The organic part consists of one MPDA^2+^ and two symmetrically independent IM^+^ cations. As depicted in Figure 2b, MPDA^2+^ moieties are located in the intralayer space and exhibit dynamic disorder. The IM^+^ cations adopt two structurally distinct sites: the fully ordered IM^+^ is placed between the layers together with MPDA^2+^, while the second, disordered IM^+^, is accommodated within the perovskite voids, where it rotates around its center of gravity. The disorder of the organic cations translates into relatively weak interactions with the inorganic part, which is reflected in the octahedra distortion parameters: the bond angle variance (σ^2^) of 19.9 deg^2^ for Pb(1) and 14.2 deg^2^ for Pb(2), and bond length distortion (Δ_*d*_) of 2.44 × 10^–4^ and 35.7 × 10^–4^ for Pb(1) and for Pb(2), respectively (both parameters calculated using Fleet’s formula).^34^ The values of σ^2^ fall within the range reported for the 3D MPDA_2_APb_4_Br_13_ analogues (A = MA, EA) but are smaller (larger) compared with MPDA_2_DMAPb_4_Br_13_ (MPDA_2_FAPb_4_Br_13_).^28,29^ On the other hand, the Δ_*d*_ value for Pb(2) is greater than that reported for Pb(2) in the 3D MPDA_2_APb_4_Br_13_ analogues (A = FA, MA, EA, and DMA), for which this value ranged from 10.3 × 10^–4^ to 30.9× 10^–4^.^28,29^ Closer inspection of the crystal structures shows that MPDA_2_IM_3_Pb_4_Br_15_ and MPDA_2_APb_4_Br_13_ compounds have very similar perovskite voids composed of corner-sharing PbBr_6_ octahedra to accommodate small-sized disordered monovalent cations. However, the presence of IM^+^ results in a change of the stoichiometry through the accommodation of two additional IM^+^ and Br^–^ ions, which removes the connection between the tetramers through edge-sharing octahedra, leading to the dimensional reduction of the 3D framework to a 2D layered architecture. The origin of this reduction is not immediately obvious, as IM^+^ possesses a smaller effective ionic radius than EA^+^ or DMA^+^,^35^ for which a 3D perovskitoid structure is stable, and thus, in principle, the size of IM^+^ is suitable for the stabilization of the 3D perovskitoid structure. We suppose that the aromaticity and cyclic shape of IM^+^ play a decisive role in destabilizing the 3D connectivity and formation of unprecedented, layered perovskite. Indeed, in contrast to MA^+^, FA^+^, DMA^+^, and EA^+^, which are known to have a strong tendency to occupy small perovskite voids in RP and DJ phases,^15,25,26^ there is not even a single example of RP or DJ phase comprising IM^+^ in the perovskite cavities. Instead, IM^+^ is known to form stable layered perovskites, as evidenced by the formation of alternating cations in the interlayer space (ACI) analogues with the formula (IM)­(A)­PbX_4_ (A = GA, methylhydrazinium (MHy), 1,2,4-triazolium (Tz)).^36–38^ Interestingly, MPDA_2_IM_3_Pb_4_Br_15_ is the first perovskite in which IM^+^ plays a dual role, i.e., as a spacer and perovskitizer cation.

**Figure 2:**
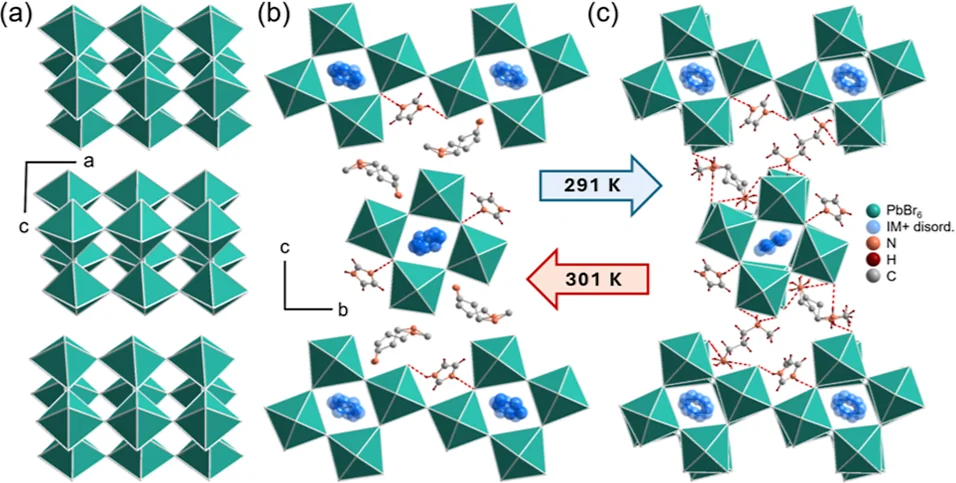
Crystal structure of MPDA_2_IM_3_Pb_4_Br_15_ in (a,b) high-temperature phase I (monoclinic, *P*2_1_/*c*) and (c) LT phase II (monoclinic, *P*2_1_/*n*). Red dashed lines illustrate N···H–Br hydrogen bonds.

Upon cooling below 290 K, MPDA_2_IM_3_Pb_4_Br_15_ undergoes a phase transition to the low-temperature (LT) phase **II** of the monoclinic, *P*2_1_/*n* symmetry. This transition is associated with a loss of translational symmetry along the [100] direction (*a*′ = 2*a*) and a corresponding increase in *Z* (the number of formula units per cell) from 2 to 4. The space groups relationship identifies the transition as klassengleiche of type IIc, with phase **II** representing a maximal isomorphic subgroup of phase **I**.^39^ The crystal structure of phase **II** is presented in Figure 2c. The dynamics of MPDA^2+^ changed from occupying a single disordered position to two crystallographically inequivalent sites, one fully ordered and the other partially disordered. In turn, the IM^+^ cations show no meaningful change relative to phase **I**, as the cation residing within the perovskite void remains disordered even upon cooling to 100 K (Figure S4b). These findings confirm the complex nature of the phase transition mechanism inferred from DSC analysis. The persistence of the partial MPDA^2+^ cation disorder and the full disorder of the cage IM^+^ in the LT phase **II** indicate that the transition is not a complete disorder-to-order process. However, the available data do not establish whether partial MPDA^2+^ cation ordering drives the symmetry breaking or results from it. The transition is therefore more appropriately described as involving coupled changes in cation ordering and crystallographic symmetry, with the measured entropy change potentially reflecting contributions from both configurational rearrangements and other structural degrees of freedom. Indeed, in the LT arrangement, all nitrogen atoms of MPDA^2+^ and ordered IM^+^ moieties are actively engaged in hydrogen bonding with Br^–^. The partial ordering of MPDA^2+^ leads to a greater distortion of the octahedral geometry, manifested in increased σ^2^ values, reaching a maximum of 27.3 deg^2^ at 270 K.

MPDA_6_GA_6_Pb_7_Br_30_Br_2_ crystallizes in the trigonal space group *P*-3*C*1. The asymmetric unit (Figure S4c) contains Pb(1)­Br_6_ and Pb(2)­Br_6_ octahedra (site symmetries 1 and 3= for Pb(1) and Pb(2), respectively), as well as one MPDA^2+^, three symmetrically independent GA^+^ cations, and one additional Br^–^ anion, present to balance the overall charge. The crystal structure of MPDA_6_GA_6_Pb_7_Br_30_Br_2_ is illustrated in Figure 3. The anionic substructure is built from units comprising eight corner-sharing PbBr_6_ octahedra, analogous to a 2 × 2 × 2 cube of the parent perovskite structure (Figure 3a). Adjacent units are connected along the *c* axis by sharing an entire Pb(2)­Br_6_ octahedra, i.e., the same octahedron occupies a corner position in one cube and the diagonally opposite corner in the neighboring one, with all six of its halides common to both units (see Figure 3a). Consequently, the Pb(2)-centered octahedra lie exactly on the *c* axis, while the surrounding Pb(1)-centered octahedra are arranged around them with a pseudohexagonal symmetry when viewed in projection along the [001] direction (Figure 3b). This connectivity generates a one-dimensional chain, in contrast to the layered topology of MPDA_2_IM_3_Pb_4_Br_15_ and 2D/3D architectures of other MPDA-based lead halides,^28,29,40,41^ which may be associated with the larger size of GA^+^. As illustrated in Figure 3b, MPDA^2+^ and two of the three symmetrically independent GA^+^ cations are located between the chains, while the remaining GA^+^ occupies the perovskite voids. All GA^+^ cations are ordered, whereas MPDA^2+^ exhibits disorder involving the CH_2_ group adjacent to the terminal NH_3_^+^ group. All nitrogen atoms participate in hydrogen bonding with Br^–^ anions, with the protonated NH_2_^+^ group of MPDA^2+^ additionally interacting with the sole nonframework Br^–^ anion. As typical for lowered dimensionality, the octahedral distortion parameters are generally reduced compared to the IM-based analogue: σ^2^ amounts to 6.62 deg^2^ and 15.3 deg^2^ for Pb(1) and Pb(2), respectively, while Δ_*d*_ equals 26.4 × 10^–4^ for Pb(1) and 0 for Pb(2) due to its symmetry.

**Figure 3:**
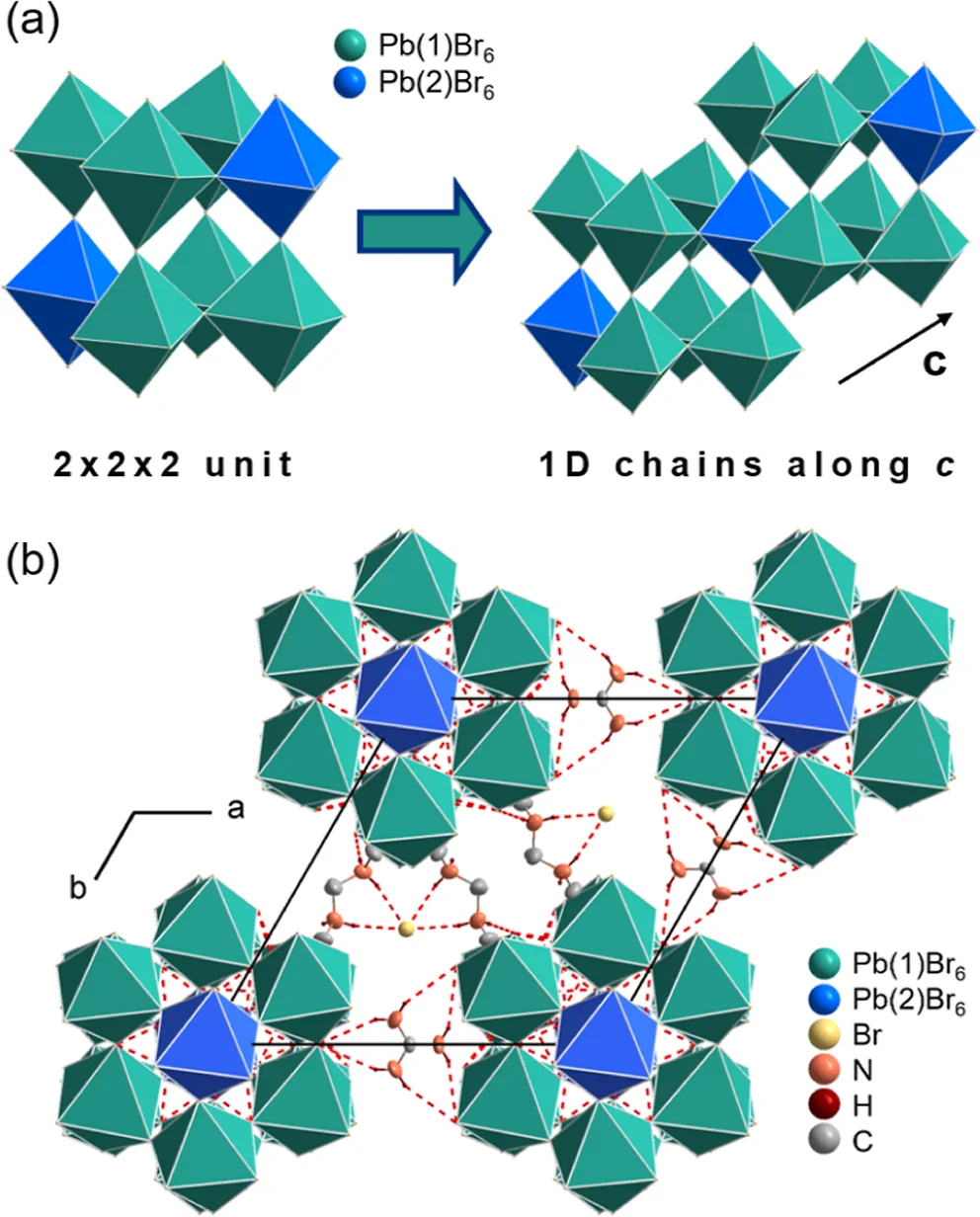
(a) Propagation scheme and (b) crystal structure of MPDA_6_GA_6_Pb_7_Br_30_Br_2_ (trigonal, *P* **3**=*c*1). Red dashed lines illustrate N···H–Br hydrogen bonds.

### Dielectric Properties

Figure 4a illustrates the temperature dependence of the real part of the complex dielectric permittivity, ε′(*T*), measured while cooling from 350 to 130 K. As shown, pronounced dielectric dispersion occurs above 300 K, with ε′ growing progressively as the frequency decreases. Both the dispersion and the ε′ values gradually diminish upon cooling toward ∼300 K, where the phase transition occurs. This structural transformation is reflected in ε′(*T*) as a considerable, frequency-independent decline in ε′. Notably, this decrease continues down to roughly 190 K and follows the exponential-like behavior, with the most pronounced changes in ε′ occurring close to the transition temperature. A similar trend can also be observed upon heating, although thermal hysteresis (typical for the phase transitions of first-order type) between the cooling and hearing cycles appears (Figure 4b). These observations indicate that the changes in dielectric permittivity associated with the phase transition have a diffuse character, in contrast with the abrupt thermal effects detected in calorimetry.

**Figure 4:**
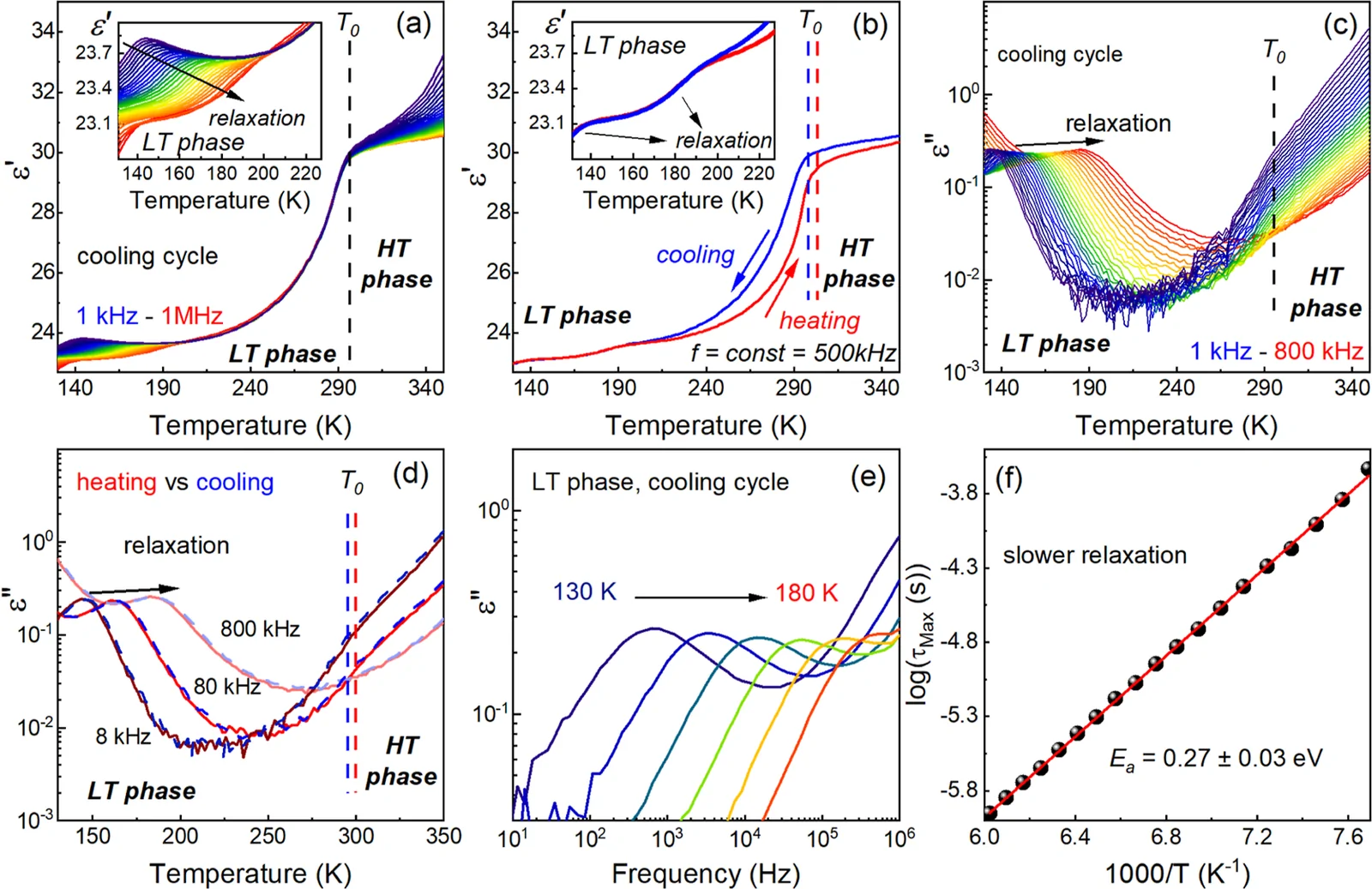
(a) ε′ measured while cooling from 350 to 130 K in the frequency range of 103–106 Hz. The inset shows the LT dielectric relaxation phenomena. (b) ε′(T) curves registered at the frequency of 500 kHz for cooling and heating cycles. The inset depicts no temperature hysteresis between cooling and heating cycles related to the relaxation phenomena. (c) Dielectric losses, ε″, measured upon cooling from 350 to 130 K in the frequency range of 103–106 Hz. (d) Comparison of the ε′′(*T*) curves obtained during cooling and heating cycles at 8 kHz, 80 kHz, and 800 kHz. (e) LT relaxation phenomena visible in the frequency-dependent dielectric loss spectra. (f) Temperature dependence of relaxation times τ_Max_ parametrized with the Arrhenius law.

Apart from the phase transition effects, the LT dielectric spectra of MPDA_2_IM_3_Pb_4_Br_15_ are characterized by clear relaxation phenomena. These processes appear in ε′(*T*) as step-like anomalies that shift toward lower temperatures with decreasing probing frequency (see insets in Figure 4a,b). Simultaneously, the dielectric relaxation manifests as temperature-sensitive bell-shaped loss peaks in the imaginary part of the complex dielectric permittivity, ε″ (see Figure 4c,d). Notably, MPDA_2_IM_3_Pb_4_Br_15_ is characterized by two LT dielectric relaxations, indicating that multiple structural units retain field-induced motional freedom in the LT phase **II**. Based on the X-ray data, the two dielectric relaxations can be most likely attributed to dynamics of MPDA^2+^ cations, which exhibit partial ordering in the LT phase **II**, and IM^+^ cations located in the perovskite cavities, which remain disordered in phase **II**. As shown in Figure 4e, one of these processes is too fast to be fully resolved below 1 MHz even at 130 K, and only its low-frequency tail is visible in the ε″(*f*) spectra. In contrast, the second relaxation process is significantly slower and appears at roughly 300 Hz at 130 K. As the temperature increases, the relaxation peak shifts toward higher frequencies, indicating that the corresponding relaxation time becomes shorter.

To characterize the LT molecular dynamics, we parametrized the dielectric ε*­(*f*) spectra with the Havriliak–Negami function (1) and determined the relaxation times τ_Max_ from the fit parameters according to eq 2. Figure 4f illustrates the resulting temperature dependence of τ_Max_. The relaxation times in the logarithm form (log_10_ τ_Max_) show a linear dependence on the inverse temperature, indicating that the slower relaxation process follows the Arrhenius behavior defined as

2 $$\tau_{\max}\left(T\right)=\tau_{0}\left(\frac{E_{a}}{k_{B}T}\right)$$

In this equation, τ_0_, *k*_B_, and *E*_a_, are the relaxation time at the limit of an infinitely high temperature, the Boltzmann constant, and the activation energy, respectively. As illustrated in Figure 4d, our experimental log­(τ_Max_) = *f*(1000/*T*) dependence fits well to the Arrhenius model with log τ_0_ ≈ −15.3 ± 0.3 and *E*_a_ ≈ 0.27 ± 0.03 eV. The obtained activation energy falls within the range commonly reported for hybrid organic–inorganic compounds featuring dynamically disordered monovalent organic cations, including IM^+^.^8,38,43^ Consequently, MPDA_2_IM_3_Pb_4_Br_15_ appears as a hybrid compound, in which some structural units remain capable of reorienting in response to the applied electric field also in the LT phase **II**.

### Optical Properties

The absorption spectra of MPDA_2_IM_3_Pb_4_Br_15_ and MPDA_6_GA_6_Pb_7_Br_30_Br_2_ registered at RT extend to 450 and 400 nm (Figure S5), with a clearly visible narrow excitonic band located at 418 nm (2.97 eV) and 377 nm (3.29 eV), respectively. The energy band gaps (*E*_g_) estimated using the Kubelka–Munk function with the Tauc relation, assuming a direct allowed electronic transition (*n* = 2), are 3.07 eV for MPDA_2_IM_3_Pb_4_Br_15_ (Figure S6) and 3.39 eV for MPDA_6_GA_6_Pb_7_Br_30_Br_2_ (Figure S7). To date, only four 3D bromide perovskitoids containing mixed MPDA and A cations have been reported, namely MPDA_2_MAPb_4_Br_13_, MPDA_2_EAPb_4_Br_13,_ MPDA_2_FAPb_4_Br_13,_ and MPDA_2_DMAPb_3_Br_10._ ^28,29^ For these perovskitoids, the *E*_g_ value ranged from 2.84 eV for FA to 3.04 eV for the DMA analogue. When comparing the *E*_g_ values of the studied compounds with those of 3D MPDA_2_APb_4_Br_13_ perovskitoids, it turns out that the band gap of 2D IM compound (1D GA compound) is only slightly (significantly) larger compared to 3D perovskitoids. In hybrid lead halides, the energy band gap typically decreases as the Pb–X bond length is reduced, and the distortion of the inorganic sublattice becomes more pronounced.^7,44,46^ It also decreases with decreasing dimensionality.^7,15,44,46^ Comparison of the crystallographic data shows that MPDA_2_IM_3_Pb_4_Br_15_ has a smaller octahedral distortion at 270 K (σ^2^ = 27.3 deg^2^ and the smallest Pb–Br–Pb angle of 162.5°, see Table S3) than MPDA_2_DMAPb_3_Br_10_ (σ^2^ = 45.9 deg^2^ and the smallest Pb–Br–Pb angle of 154.9°).^29^ Therefore, lowering of dimensionality to 2D when DMA is replaced by IM is partially compensated by smaller octahedral distortion, leading to a slight increase of *E*_g_. MPDA_6_GA_6_Pb_7_Br_30_Br_2_ also exhibits smaller octahedral distortion at 270 K (σ^2^ = 6.62 + 15.3 deg^2^ and the smallest Pb–Br–Pb angle of 162.0°, Table S4) than MPDA_2_DMAPb_3_Br_10_. In this case, large widening of the band gap can be, therefore, attributed to a pronounced decrease of dimensionality.

At 80 K, both investigated compounds exhibit intense PL that covers the whole visible spectral range (Figure 5a). The PL of MPDA_2_IM_3_Pb_4_Br_15_ is dominated by the narrow bands centered at 413 and 420 nm, which are associated with the recombination of free (FE) and bound (bound exciton (BE)) excitons, respectively (Figure S5a). Additionally, a significantly weaker broad emission centered around 600 nm is observed. The very large Stokes shift (≈0.9 eV) and large full width at half-maximum (fwhm) of 0.56 eV are typical for self-trapped excitation (STE) emission or structural defect recombination occurring in low-dimensional lead halides.^4,7,14,15,22^ Such broad STE emission is commonly observed in low-dimensional hybrid lead halides and is generally attributed to strong exciton–phonon coupling, often accompanied by pronounced out-of-plane octahedral tilting of the inorganic octahedra.^4,15,47,48^ Much weaker intensity of the broadband emission observed in 2D MPDA_2_IM_3_Pb_4_Br_15_ compared to that reported in 3D MPDA_2_DMAPb_3_Br_10_ is consistent with smaller octahedral distortion of the former compound. On the other hand, PL of 2D MPDA_2_IM_3_Pb_4_Br_15_ is like that reported for 3D MPDA_2_EAPb_4_Br_13_, which has significantly weaker octahedral distortion than its DMA analogue.^29^ In contrast, PL of MPDA_6_GA_6_Pb_7_Br_30_Br_2_ consists of a single intense band spanning from approximately 450 to 800 nm (fwhm = 0.5 eV), characteristic of STE-related recombination. The lack of FE-related emission in MPDA_6_GA_6_Pb_7_Br_30_Br_2_ despite its slightly lower octahedral distortion compared to MPDA_2_IM_3_Pb_4_Br_15_ can be most likely attributed to lowering dimensionality to 1D. The difference in the spectral position and origin of the PL leads to markedly different emission colors. For the IM analogue, the chromaticity coordinates of (*x*, *y*) = (0.37, 0.32) fall within the white-light region of the CIE 1931 chromaticity diagram and emit pinkish-white light, while the GA sample exhibits greenish-yellow emission with (*x*, *s*) = (0.47, 0.50) (Figure 5b). A similar type and color of PL have been reported recently for MPDA_2_DMAPb_4_Br_13_, which exhibited yellow emission at 80 K.^29^

**Figure 5:**
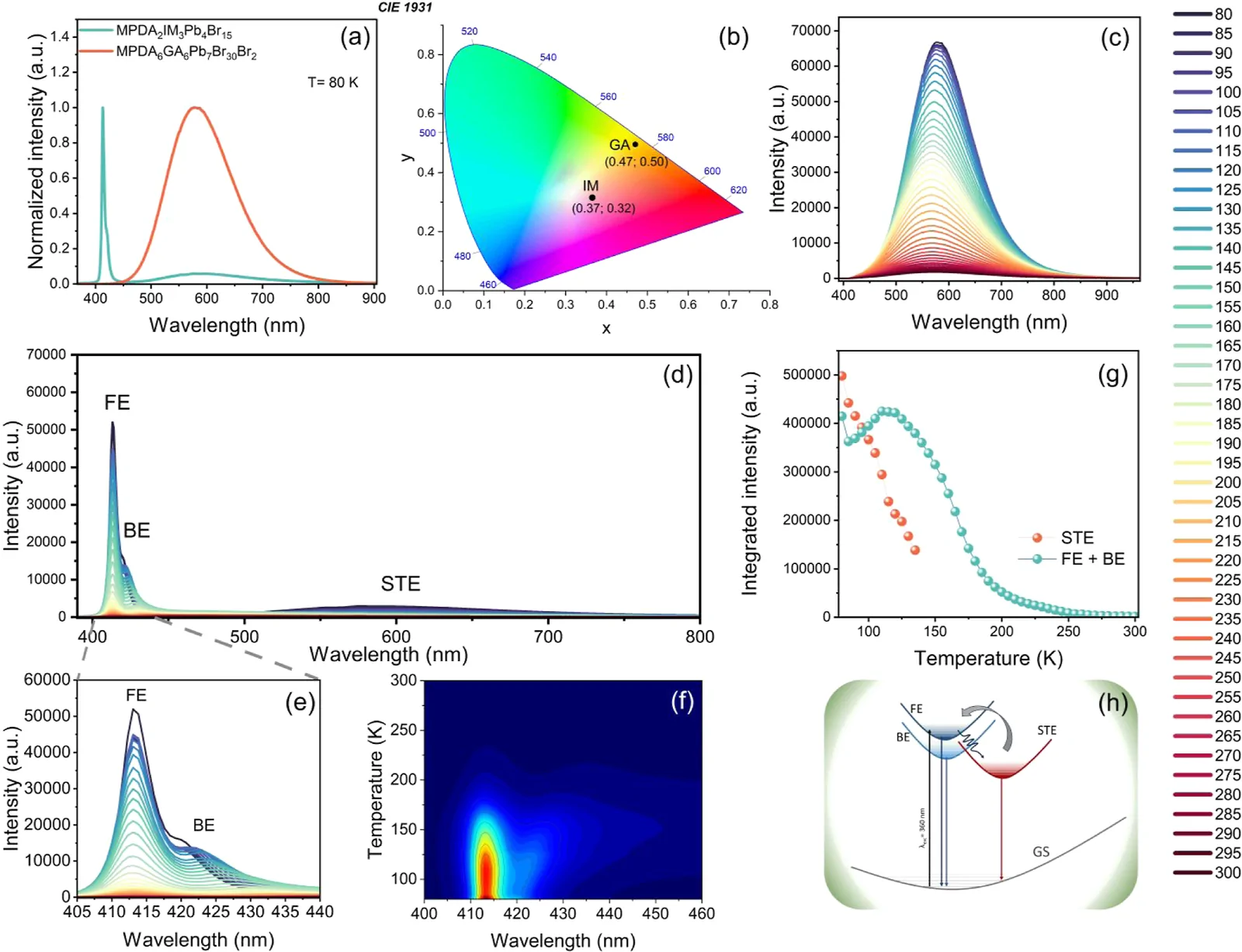
(a) Comparison of the normalized emission spectra of MPDA_2_IM_3_Pb_4_Br_15_ and MPDA_6_GA_6_Pb_7_Br_30_Br_2_ at 80 K. (b) CIE diagram with marked chromaticity coordinates *x*, *y*. (c) Temperature-dependent PL spectra of the MPDA-GA sample. (d) PL spectra as a function of temperature for the MPDA_2_IM_3_Pb_4_Br_15_ analogue. (e) Closer look at the temperature plot showing the FE and BE bands. (f) Map of emission intensity as a function of temperature for the FE and BE bands. (g) Variation in the integral intensity of the FE + BE and STE bands for MPDA_2_IM_3_Pb_4_Br_15_. (h) Configuration diagram illustrating the mechanism of PL processes and energy transfer from STE levels to FE and BE levels stimulated by temperature.

Temperature-dependent spectra recorded over the range 80–300 at 5 K intervals showed that the emission of MPDA_6_GA_6_Pb_7_Br_30_Br_2_ decays slowly with increasing temperature (Figures 5c and Figure S8). The activation energy for thermal quenching of PL is 153 meV (Figure S9), and the *T*_0.5_ value is as high as 195 K. It follows that the MPDA_6_GA_6_Pb_7_Br_30_Br_2_ emission is much more stable than the emission of previously reported 3D MPDA_2_APb_3_Br_10_ perovskitoids, for which *T*_0.5_ have not exceeded 100 K.^29^ Closer inspection of the PL data shows that the PL band exhibits a slight band shift toward the blue on heating. However, this shift is so small that it leads to minor changes in the CIE *x*, *y* parameters, and the observed emission color remains greenish-yellow across the entire temperature range (Figure S10).

The temperature-dependent PL measurements of MPDA_2_IM_3_Pb_4_Br_15_ reveal a pronounced redistribution of the excited-state population among the STE, BE, and FE states. Upon closer inspection, it turns out that as the temperature increases, the STE emission decays very rapidly and disappears completely at 135 K (Figure 5g). In parallel, the integrated intensity of the FE + BE emission increases between 85 and 110 K, indicating a thermally activated population transfer from the STE toward the less localized FE and BE states (Figure 5h). The subsequent near-constant intensity observed between 110 and 120 K suggests that the thermally activated depopulation of the STE state is approximately balanced by competing relaxation and recombination pathways. Above 125 K, the FE + BE emission undergoes a gradual thermal quenching (*E*_a_ = 152 meV, Figure S11), consistent with the onset of thermally activated nonradiative recombination. Interestingly, energies of the PL bands exhibit pronounced temperature dependence (Figure S12), i.e., while the FE emission remains nearly unchanged in energy over the investigated temperature range, the BE band gradually red-shifts and the STE band exhibits a noticeable blue shift upon heating. This distinct behavior indicates that the three emissive states respond differently to the thermally induced lattice dynamics. The nearly temperature-independent FE energy is consistent with its predominantly band-edge character, whereas the red shift of the BE emission points to a stronger sensitivity of the localized excitonic state to the local structural environment and exciton–phonon coupling.^4,15,47,48^ The blue shift of the STE band can be rationalized by the progressive destabilization of the strongly lattice-relaxed STE configuration upon thermal activation, which facilitates detrapping toward higher-energy FE/BE states.^4,14,48,49^ Such behavior is consistent with a thermally driven redistribution between localized and delocalized excitonic configurations rather than with independent thermal quenching of the individual emissive states.

As a consequence, the emission chromaticity exhibits a pronounced temperature-dependent evolution (Figure S13). At low temperatures (80–100 K), the emission is located close to the white region of the CIE 1931 chromaticity diagram and can be visually perceived as white light with a subtle pinkish-purplish tone. Upon increasing the temperature to 105–125 K, the chromaticity progressively shifts toward the purple and purplish-blue region (130–175 K). Further heating to 180–275 K induces a gradual shift toward the greenish-blue and bluish-green region. At higher temperatures (280–300 K), the chromaticity moves back toward the near-white region, with the emission being perceived as white light with an increasingly pronounced yellowish-green and yellow-green tint.

## Conclusions

Single crystals of two mixed-cation lead bromides comprising diammonium MPDA and monoammonium IM^+^ or GA^+^ cations were successfully grown. X-ray diffraction studies revealed that MPDA_2_IM_3_Pb_4_Br_15_ crystallizes in a 2D layered structure with unprecedented layers built up of the corner-sharing octahedra organized into tetrameric units, which are also connected by corners. The formed perovskite voids are occupied by disordered IM^+^ cations. Thus, MPDA_2_IM_3_Pb_4_Br_15_ is the first example of a hybrid lead halide perovskite comprising IM^+^ cations in the perovskite voids. The layers are separated by MPDA^2+^ and IM^+^, which are partially ordered at the LTs. However, temperature activates significant motions of MPDA^2+^ cations, leading to the structural phase transition at *T*_1_ = 301 K (291 K) during heating (cooling), respectively. This phase transition leads to a pronounced dielectric anomaly. The GA-based analogue, MPDA_6_GA_6_Pb_7_Br_30_Br_2_, crystallizes in a 1D structure, which comprises chains composed of corner-sharing PbBr6 units separated by GA^+^ cations. In contrast to the IM-based perovskites, the GA-based perovskitoid does not exhibit any phase transitions.

Both compounds exhibit PL, broadband for MPDA_6_GA_6_Pb_7_Br_30_Br_2_ and composed of both narrow and broad bands in the case of MPDA_2_IM_3_Pb_4_Br_15_. PL of MPDA_6_GA_6_Pb_7_Br_30_Br_2_ is very stable, and its color remains greenish-yellow across the entire temperature range. In contrast to the GA-based perovskitoid, PL of MPDA_2_IM_3_Pb_4_Br_15_ exhibits strong thermochromism with color changing from white at very low temperatures via the purple and purplish-blue region in the 105–175 K range toward greenish blue and bluish green in the 180–275 K region and finally back toward the near-white region near RT. Overall, this work shows that the mixing of diammonium and monoammonium cations is a very efficient strategy for controlling the dimensionality and dielectric and optical properties of hybrid lead halides.

## Supplementary Material



## Notes

Additional experimental details include photos of the grown crystals, powder X-ray diffraction diffractograms, DSC traces, basic structural units of MPDA_2_IM_3_Pb_4_Br_15_, diffuse reflection spectra, Tauc plots, temperature-dependent PL intensities and bands’ positions, CIE diagrams, and selected structural parameters (PDF)

## References

[1] Saparov B., Mitzi D. B. (2016). Organic-Inorganic Perovskites: Structural Versatility
for Functional Materials Design. Chem. Rev..

[2] Metcalf I., Sidhik S., Zhang H., Agrawal A., Persaud J., Hou J., Even J., Mohite A. D. (2023). Synergy of 3D and 2D Perovskites
for Durable, Efficient Solar Cells and Beyond. Chem. Rev..

[3] Huang Y., Li Y., Lim E. L., Kong T., Zhang Y., Song J., Hagfeldt A., Bi D. (2021). Stable Layered Perovskite Solar Cells
with an Efficiency over 19% via Multifunctional Interfacial Engineering. J. Am. Chem. Soc..

[4] Smith M. D., Connor B. A., Karunadasa H. I. (2019). Tuning the Luminescence of Layered
Halide Perovskites. Chem. Rev..

[5] Kar S., Jamaludin N. F., Yantara N., Mhaisalkar S. G., Leong W. L. (2021). Recent Advancements
and Perspectives on Light Management
and High Performance in Perovskite Light-Emitting Diodes. Nanophotonics.

[6] Han Y., Cui B. B. (2021). Low-dimensional
Metal Halide Perovskite Materials:
Structure Strategies and Luminescence Applications. Adv. Sci..

[7] Mączka M., Gagor A., Stefanska A., Hanuza J., Kucharska E., Zareba J. K. (2025). Divalent Methylhydrazinium-An
Ultrasmall Organic Cation
for Construction of Hybrid Perovskites. Chem.
Mater..

[8] Smółka S., Mączka M., Drozdowski D., Stefańska D., Gągor A., Sieradzki A., Zaręba J. K., Ptak M. (2022). Effect of Dimensionality
on Photoluminescence and Dielectric Properties
of Imidazolium Lead Bromides. Inorg. Chem..

[9] Gilley I. W., Wiggins T. E., Sargent E. H., Kanatzidis M. G. (2025). Perovskitoids
as Functional Materials. Acc. Chem. Res..

[10] Stoumpos C. C., Malliakas C. D., Kanatzidis M. G. (2013). Semiconducting Tin and Lead Iodide
Perovskites with Organic Cations: Phase Transitions, High Mobilities,
and Near-Infrared Photoluminescent Properties. Inorg. Chem..

[11] Petrosova H. R., Kucheriv O. I., Shova S., Gural’skiy I. A. (2022). Aziridinium
Cation Templating 3D Lead Halide Hybrid Perovskites. Chem. Commun..

[12] Maczka M., Ptak M., Gagor A., Zareba J., Liang X., Balciunas S., Semenikhin O. A., Kucheriv O. I., Gural’skiy I. A., Shova S., Walsh A., Banys J., Šimėnas M. (2023). Phase Transitions,
Dielectric Response, and Nonlinear Optical Properties of Aziridinium
Lead Halide Perovskites. Chem. Mater..

[13] Li W., Wang Z., Deschler F., Gao S., Friend R. H., Cheetham A. K. (2017). Chemically Diverse and Multifunctional
Hybrid Organic–Inorganic
Perovskites. Nat. Rev. Mater..

[14] Maczka M., Sobczak S., Roszak K., Vasconcelos D. L. M., Dybała F., Herman A. P., Kudrawiec R., Katrusiak A., Freire P. T. C. (2025). Pressure-Induced Detrapping from
Self-Trapped Excitons to Free Excitons Toward Enhanced Emission and
Piezochromism in Ruddlesden-Popper (110)-Oriented Perovskite. ACS Appl. Mater. Interfaces.

[15] Maczka M., Stefańska D., Ptak M., Gagor A., Fedoruk-Piskorska K., Smółka S., Zareba J. K., Sieradzki A. (2025). Dion–Jacobson
Lead Bromide Perovskites with 1,4-Butanediammonium Cations: Lattice
Dynamics, Phase Transitions, Dielectric Switching, and Optical Properties. Inorg. Chem..

[16] Dohner E. R., Hoke E. T., Karunadasa H. I. (2014). Self-Assembly
of Broadband White-Light
Emitters. J. Am. Chem. Soc..

[17] Tang J., He B., Kuang K., Li M., Cao S., Yu Z., He Y., Chen J. (2024). Bulk Photovoltaic Effect in Polar 3D Perovskitoid Enables
Self-Powered Polarization-Sensitive Photodetection. Small.

[18] Li X., He Y., Kepenekian M., Guo P., Ke W., Even J., Katan C., Stoumpos C. C., Schaller R. D., Kanatzidis M. G. (2020). Three-Dimensional
Lead Iodide Perovskitoid Hybrids with High X-ray Photoresponse. J. Am. Chem. Soc..

[19] Tang Y.-Y., Liu Y.-H., Peng H., Deng B.-B., Cheng T.-T., Hu Y.-T. (2020). Three-Dimensional Lead Bromide Hybrid Ferroelectric Realized by Lattice
Expansion. J. Am. Chem. Soc..

[20] Zhang H.-Y., Song X.-J., Cheng H., Zeng Y.-L., Zhang Y., Li P.-F., Liao W.-Q., Xiong R.-G. (2020). A Three-Dimensional
Lead Halide Perovskite-Related Ferroelectric. J. Am. Chem. Soc..

[21] Li X., Kepenekian M., Li L., Dong H., Stoumpos C. C., Seshadri R., Katan C., Guo P., Even J., Kanatzidis M. G. (2022). Tolerance Factor for Stabilizing
3D Hybrid Halide Perovskitoids
Using Linear Diammonium Cations. J. Am. Chem.
Soc..

[22] Cao S., Kuang K., He B., Tang J., Yu Z., Li M., He Y., Chen J. (2024). Methylated diammonium spacer modulated
three-dimensional lead bromide perovskitoid hybrids with distinct
photoconductivity anisotropy. Inorg. Chem. Front..

[23] Zhang Y., Abdi-Jalebi M., Larson B. W., Zhang F. (2024). What Matters for the
Charge Transport of 2D Perovskites?. Adv. Mater..

[24] Siwach P., Sikarwar P., Halpati J. S., Chandiran A. K. (2022). Design
of Above-Room-Temperature Ferroelectric Two-Dimensional Layered Halide
Perovskites. J. Mater. Chem. A.

[25] Simenas M., Gagor A., Banys J., Maczka M. (2024). Phase Transitions and
Dynamics in Mixed Three- and Low-Dimensional Lead Halide Perovskites. Chem. Rev..

[26] Mączka M., Gągor A., Popanda B., Ptak M., Stefańska D., Fedoruk-Piskorska K., Zaręba J. K., Sieradzki A. (2026). Isopropylammonium
as a Dual-Role Cation: Spacer and Perovskitizer in Ruddlesden–Popper
Lead Bromides. Inorg. Chem..

[27] Gilley I. W., Kwon H. W., Liu C., Yang Y., Huang C., Wan H., Bati A. S. R., Oriel E. H., Kepenekian M., Vishal B., Zeiske S., Bayikadi K. S., Wiggins T. E., Vasileiadou E. S., Chen B., Schaller R. D., Even J., De Wolf S., Sargent E. H., Kanatzidis M. G. (2025). Combining
Organic Cations of Different Sizes Grants Improved Control over Perovskitoid
Dimensionality and Bandgap. J. Am. Chem. Soc..

[28] Ji C., Zhu T., Fan Y., Li Z., Liu X., Li L., Sun Z., Luo J. (2022). Localized Lattice Expansion of FAPbBr_3_ to
Design a 3D Hybrid Perovskite for Sensitive Near-Infrared Photodetection. Angew. Chem., Int. Ed..

[29] Mączka M., Drozdowski D., Stefanska D., Popanda B., Anilkumar V., Prochowicz D. (2026). 3D Hybrid Perovskitoid Single Crystals Constructed
with Di- and Monoammonium Cations for Light-Emitting and Photodetection. Chem. Mater..

[30] Kieslich G., Sun S., Cheetham A. K. (2014). Solid-State
Principles Applied to Organic–Inorganic
Perovskites: New Tricks for an Old Dog. Chem.
Sci..

[31] Dolomanov O. V., Bourhis L. J., Gildea R. J., Howard J. A. K., Puschmann H. (2009). OLEX2: A Complete
Structure Solution, Refinement and Analysis Program. J. Appl. Crystallogr..

[32] Sheldrick G. M. (2015). SHELXT
– Integrated Space-Group and Crystal-Structure Determination. Acta Crystallogr. A.

[33] Sheldrick G. M. (2015). Crystal
Structure Refinement with SHELXL. Acta Crystallogr.
C.

[34] Fleet M. E. (1976). Distortion
Parameters for Coordination Polyhedra. Mineral.
Mag..

[35] Kieslich G., Sun S., Cheetham A. K. (2015). An Extended
Tolerance Factor Approach for Organic–Inorganic
Perovskites. Chem. Sci..

[36] Guo Y. Y., McNulty J. A., Mica N. A., Samuel I. D. W., Slawin A. M. Z., Bühl M., Lightfoot P. (2019). Structure-Directing Effects in (110)-Layered
Hybrid Perovskites Containing Two Distinct Organic Moieties. Chem. Commun..

[37] Guo Y. Y., Yang L. J., Biberger S., McNulty J. A., Li T., Schötz K., Panzer F., Lightfoot P. (2020). Structural
Diversity in Layered Hybrid Perovskites, A_2_PbBr_4_ or AA′PbBr_4_, Templated by Small Disc-Shaped Amines. Inorg. Chem..

[38] Drozdowski D., Fedoruk K., Kabanski A., Maczka M., Sieradzki A., Gagor A. (2023). Broadband Yellow and
White Emission from Large Octahedral Tilting
in (110)-oriented Layered Perovskites: Imidazolium-Methylhydrazinium
Lead Halides. J. Mater. Chem. C.

[39] International Tables for Crystallography; Hahn, T. , Ed.; Chester: England, 2006; Vol. A.

[40] Fan C. C., Liu C. D., Liang B. D., Jin M. L., Ju T. Y., Chai C. Y., Han X. B., Zhang W. (2023). A Two-Dimensional Hybrid
Lead Bromide Ferroelectric Semiconductor with an Out-of-Plane Polarization. Inorg. Chem..

[41] Mączka M., Drozdowski D., Fedoruk-Piskorska K., Kudrawiec J., Popanda B., Zaręba J. K., Bartoszewicz R., Sieradzki A. (2026). Two-Dimensional Dion-Jacobson Ferrroelectric
Semiconductors
with an Out-of-Plane Polarization and Ultraviolet Photoluminescence. Chem. Mater..

[43] Mączka M., Sobczak S., Ptak M., Smólka S., Fedoruk K., Dybała F., Herman A. P., Paraguassu W., Zaręba J. K., Kudrawiec R., Sieradzki A., Katrusiak A. (2024). Revisiting
a (001)-oriented Layered Lead Chloride Templated
by 1,2,4-Triazolium: Structural Phase Transitions, Lattice Dynamics
and Broadband Photoluminescence. Dalton Trans..

[44] Mao L., Stoumpos C. C., Kanatzidis M. G. (2019). Two-Dimensional Hybrid Halide Perovskites:
Principles and Promises. J. Am. Chem. Soc..

[46] Li X., Fu Y., Pedesseau L., Guo P., Cuthriell S., Hadar I., Even J., Katan C., Stoumpos C. C., Schaller R. D., Harel E., Kanatzidis M. G. (2020). Negative
Pressure Engineering with Large Cage Cations in 2D Halide Perovskites
Causes Lattice Softening. J. Am. Chem. Soc..

[47] Febriansyah B., Borzda T., Cortecchia D., Neutzner S., Folpini G., Koh T. M., Li Y., Mathews N., Petrozza A., England J. (2020). Metal Coordination
Sphere Deformation Induced Highly
Stokes-Shifted, Ultra Broadband Emission in 2D Hybrid Lead-Bromide
Perovskites and Investigation of Its Origin. Angew. Chem., Int. Ed..

[48] Smith M. D., Jaffe A., Dohner E. R., Lindenberg A. M., Karunadasa H. I. (2017). Structural Origins of Broadband Emission from Layered
Pb-Br Hybrid Perovskites. Chem. Sci..

[49] DuBose J. T., Kamat P. V. (2023). How Pendant Groups Dictate Energy and Electron Transfer
in Perovskite–Rhodamine Light Harvesting Assemblies. J. Am. Chem. Soc..

