# Supplementary material for: Modulation of Mixed-Cation Lead Halide Crystal Structures and Properties by Changing Small Size Cation
Source: Inorg Chem. 2026 Oct 28;65(44):26132–40. doi: 10.1021/acs.inorgchem.6c04471 (PMC13753726; doi:10.1021/acs.inorgchem.6c04471)
Supplement: Supplementary file 1 [file ic6c04471_si_001.pdf]

Supporting Information for

Modulation of Mixed-Cation Lead Halide Crystal Structures and Properties by Changing Small Size Cation

Mirosław Mączka,*[,a] Dawid Drozdowski,[a] Andrzej Nowok,[b] Dagmara Stefańska,[a] Barbara Popanda,[a] and Adam Sieradzki[b]

[a]*Institute of Low Temperature and Structure Research, Polish Academy of Sciences, ul. Okólna 2, 50-422 Wrocław, Poland*

[b]*Department of Experimental Physics, Wrocław University of Science and Technology, Wybrzeże Wyspiańskiego 27, 50-370, Wrocław, Poland*

e-mail: m.maczka@intibs.pl

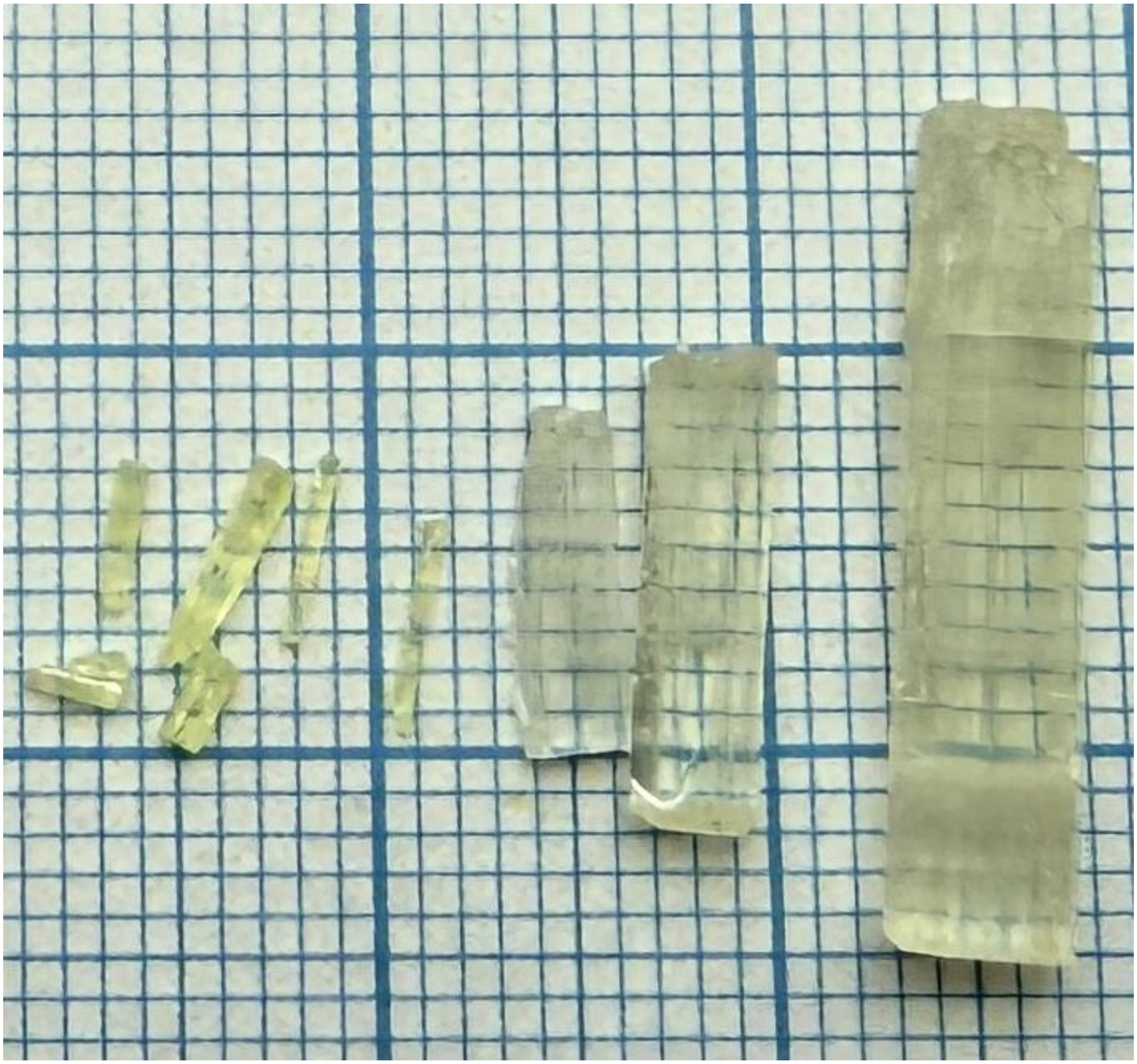

Figure S1. Images of the grown $MPDA_2IM_3Pb_4Br_{15}$ (left) and $MPDA_6GA_6Pb_7Br_{30}Br_2$ (right) crystals.

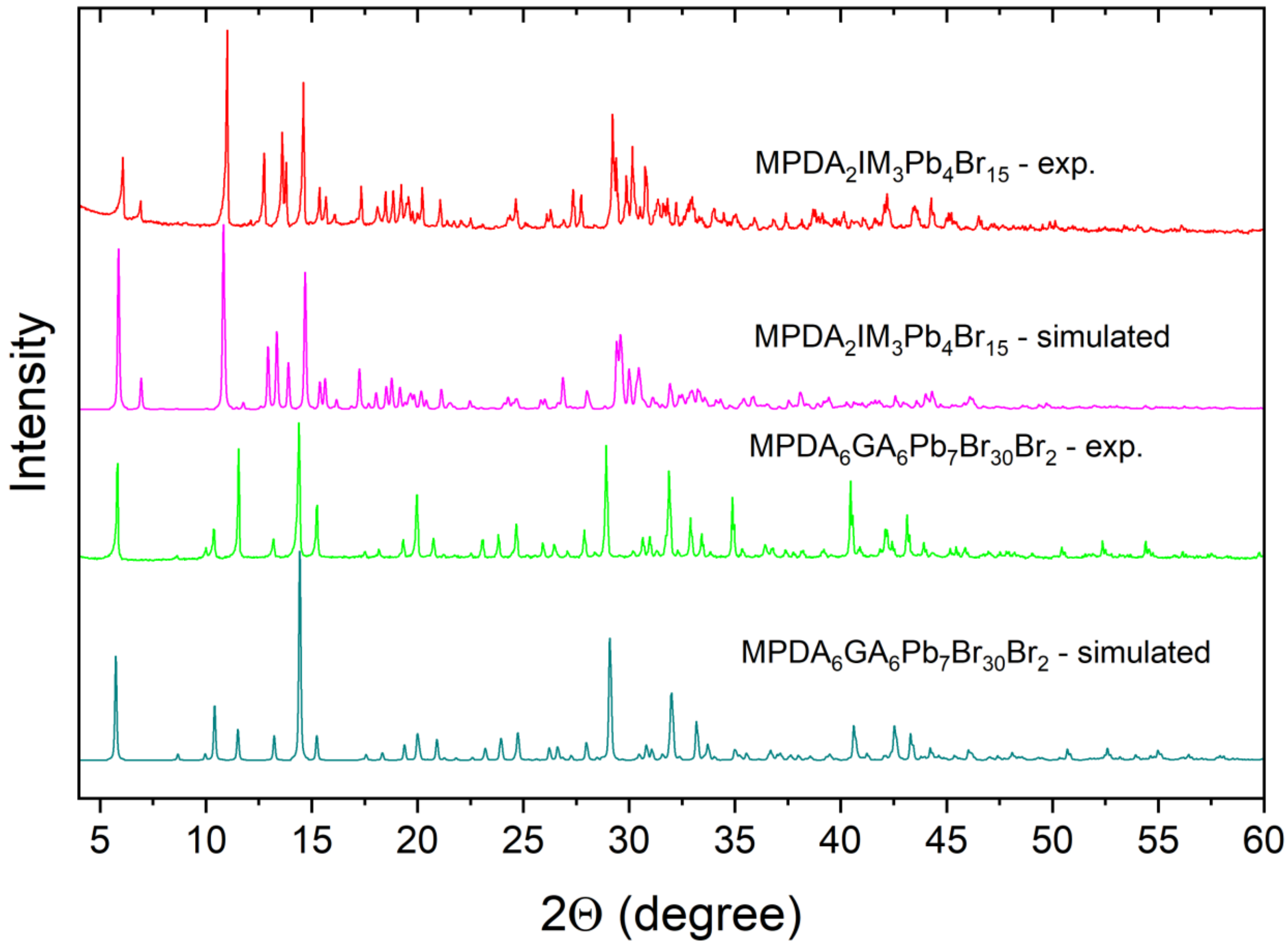

Figure S2. Experimental and simulated powder XRD patterns of $MPDA_2IM_3Pb_4Br_{15}$ and $MPDA_6GA_6Pb_7Br_{30}Br_2$.

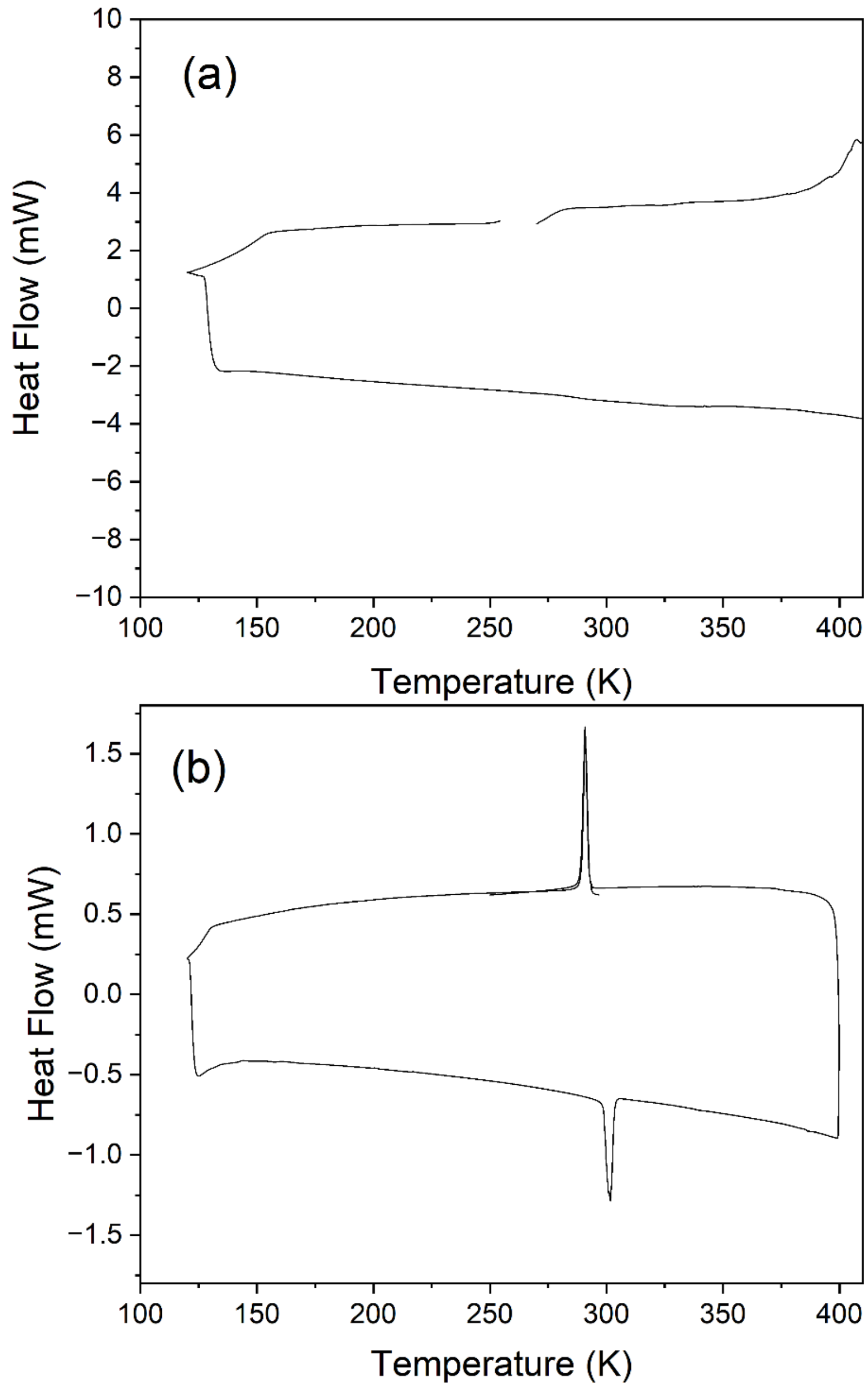

Figure S3. DSC traces for (a) $MPDA_6GA_6Pb_7Br_{30}Br_2$ and (b) $MPDA_2IM_3Pb_4Br_{15}$ in heating and cooling mode.

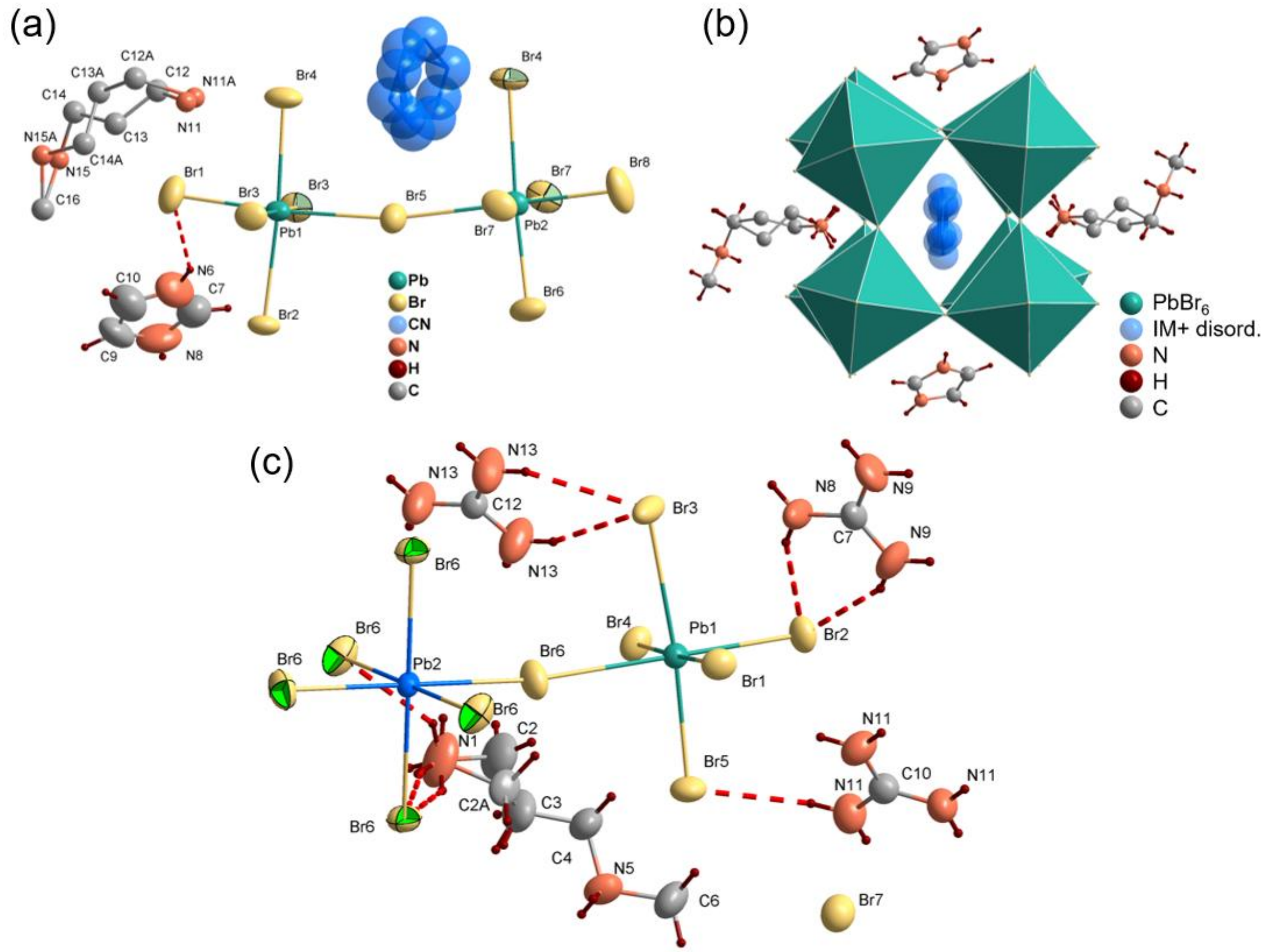

Figure S4. (a) Basic structural units of $MPDA_2IM_3Pb_4Br_{15}$ in high-temperature phase **I** (monoclinic, $P2_1/c$) at 310 K. (b) Fragment of $MPDA_2IM_3Pb_4Br_{15}$ crystal structure in low-temperature phase **II** (monoclinic, $P2_1/n$) at 100 K, showing the preservation of characteristics of organic dynamics versus 270 K, i.e., disorder of IM^+ localized in the perovskite voids and partial disorder of $MPDA^{2+}$ cations. (c) Basic structural units of $MPDA_6GA_6Pb_7Br_{30}Br_2$ at 295 K (trigonal, $P\overline{3}c1$). Bromines generated with symmetry elements in (a, c) are presented as octants. Red dashed lines denote N···H–Br hydrogen bonds.

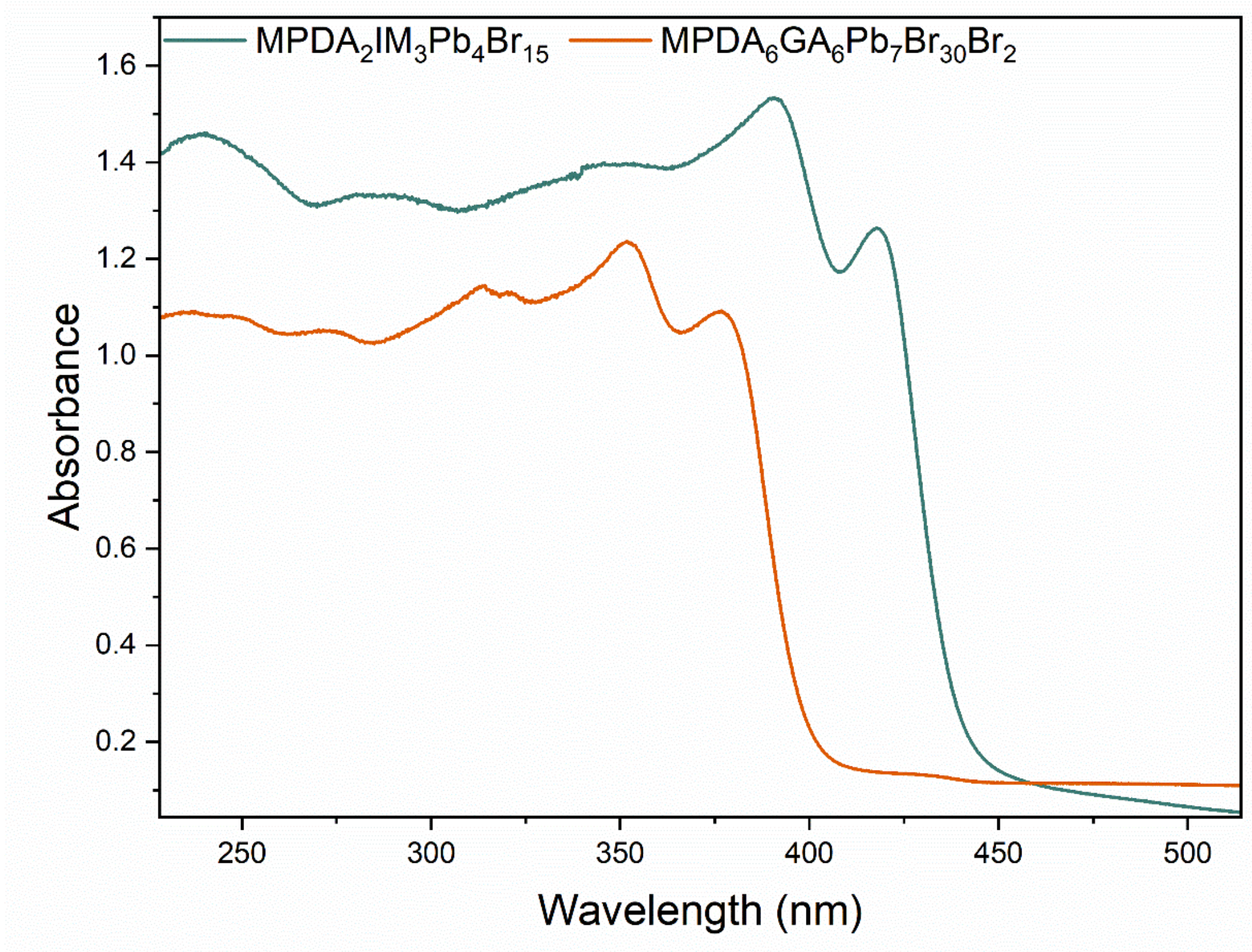

Figure S5. Diffuse reflectance-derived absorption spectra of $MPDA_2IM_3Pb_4Br_{15}$ and $MPDA_6GA_6Pb_7Br_{30}Br_2$ registered at RT.

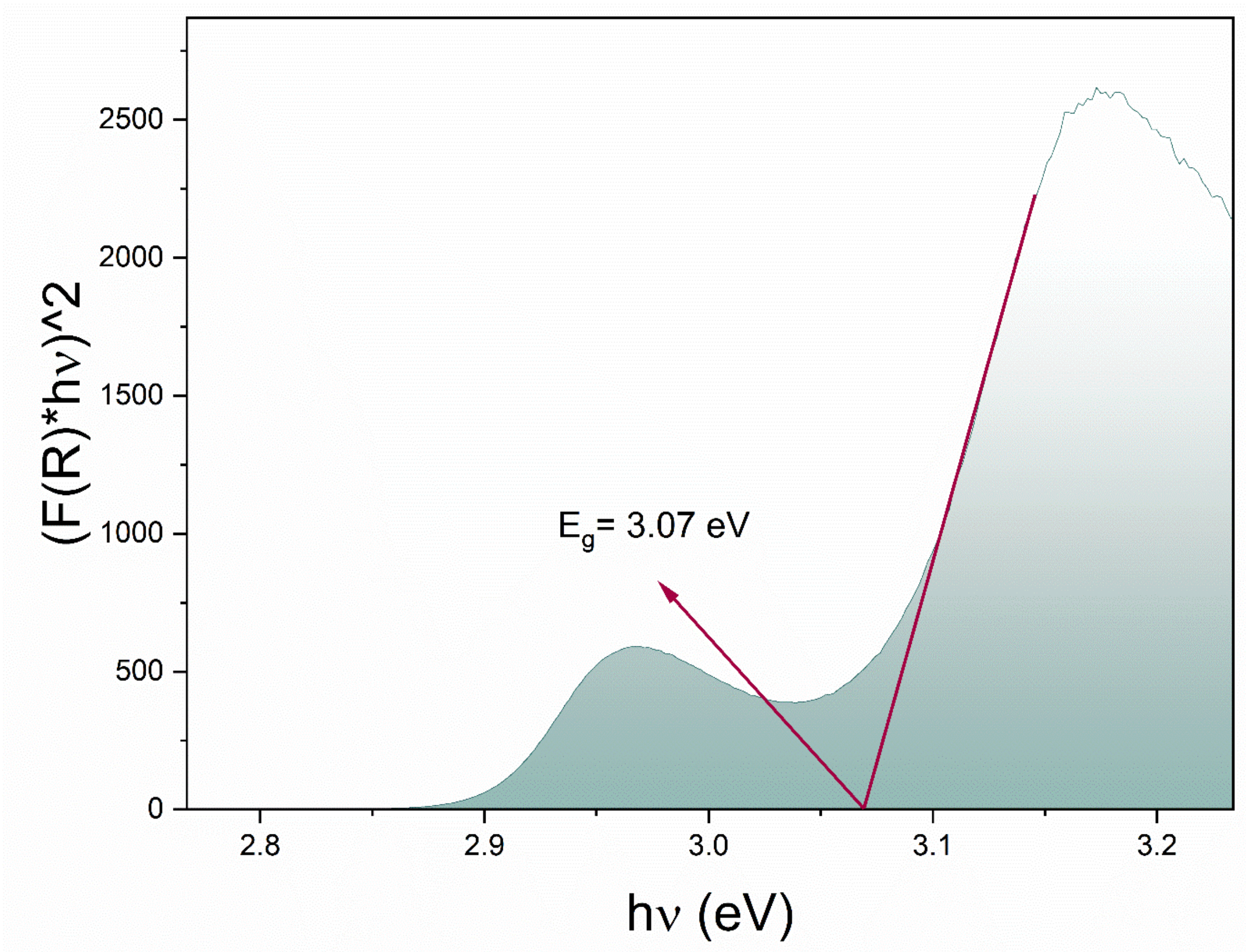

Figure S6. Energy bang gap of $MPDA_2IM_3Pb_4Br_{15}$ estimated by x-axis intersection points of the linear fits of the Tauc plots.

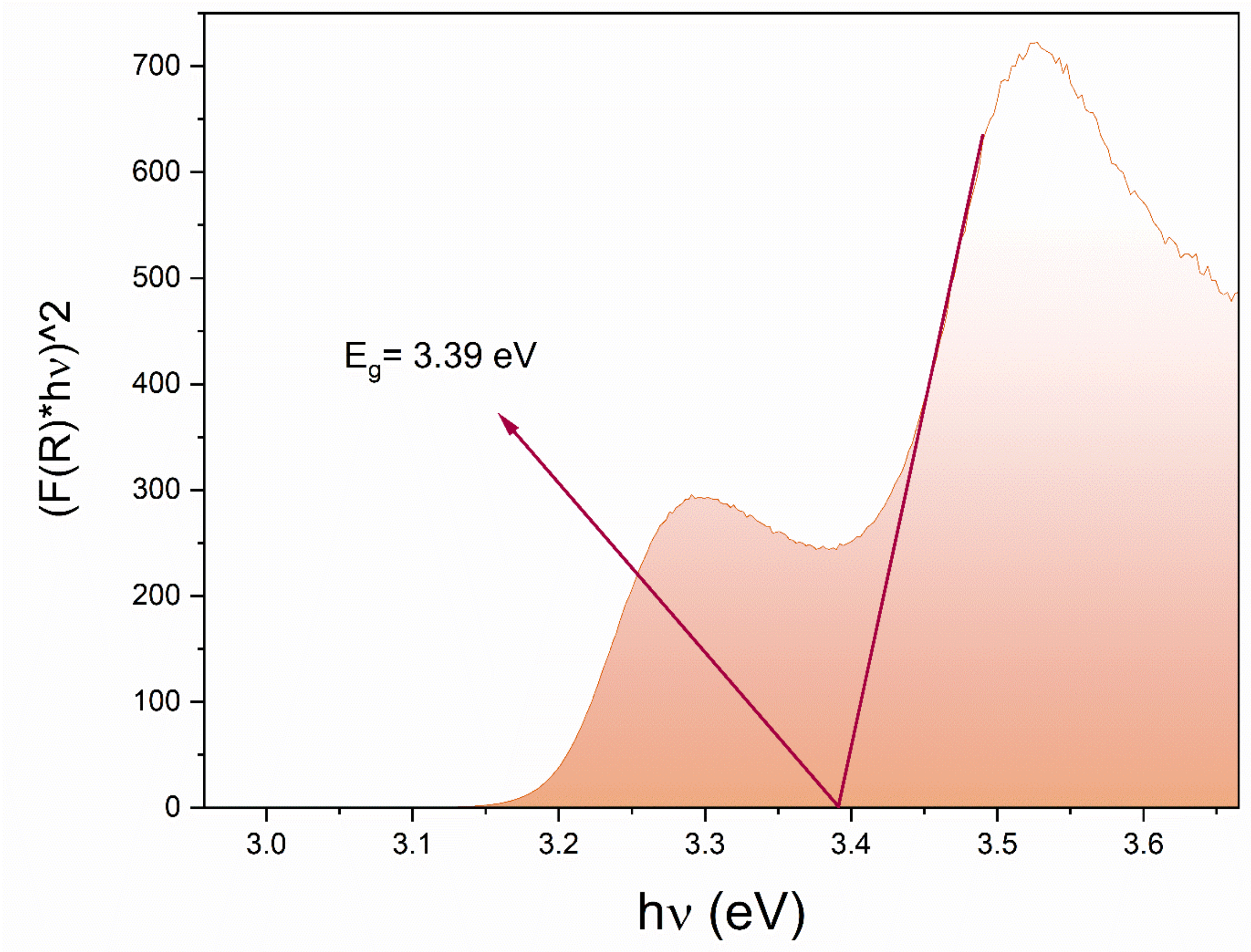

Figure S7. Energy bang gap of $MPDA_6GA_6Pb_7Br_{30}Br_2$ estimated by x-axis intersection points of the linear fits of the Tauc plots.

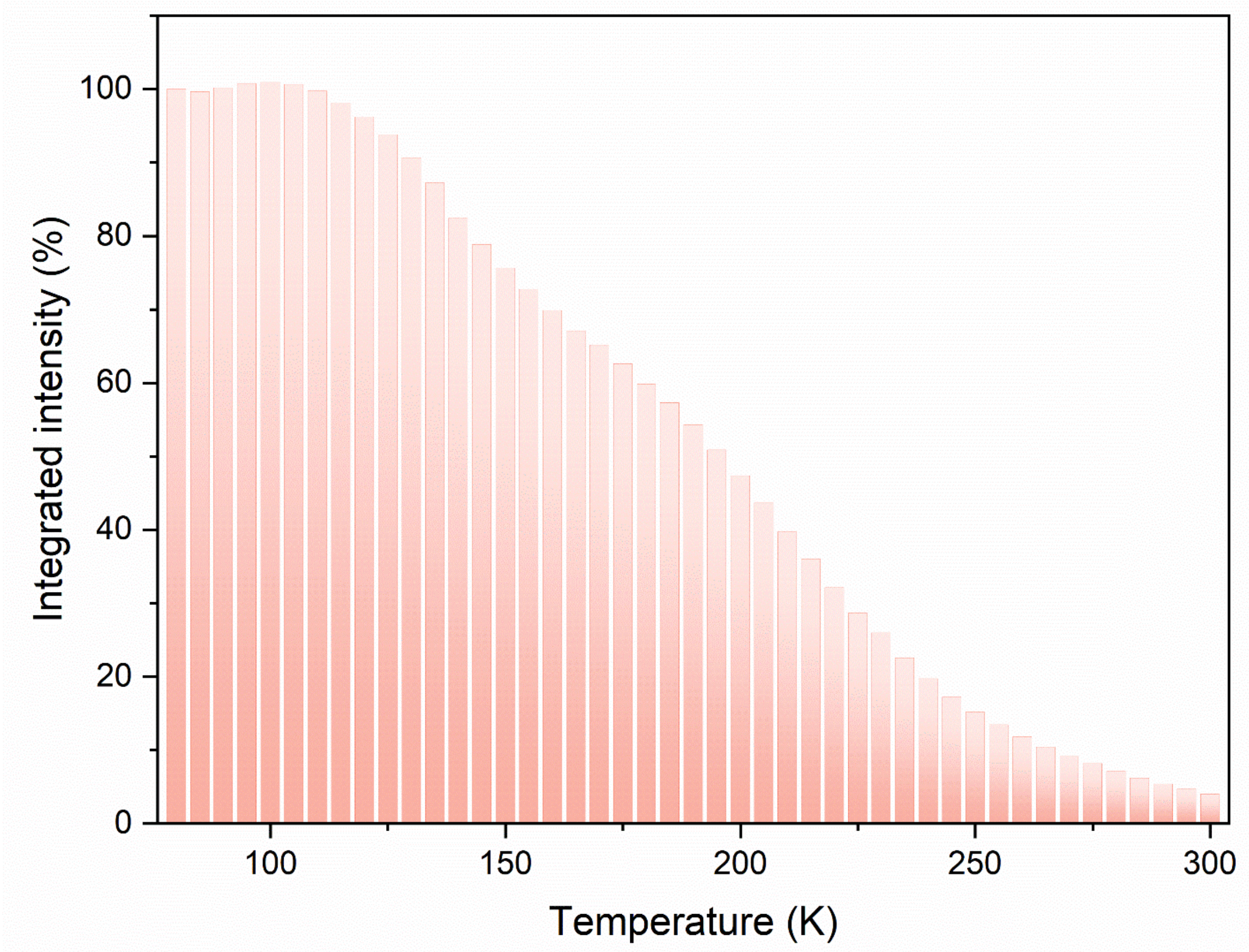

Figure S8. Integrated emission intensity in function of temperature of $MPDA_6GA_6Pb_7Br_{30}Br_2$.

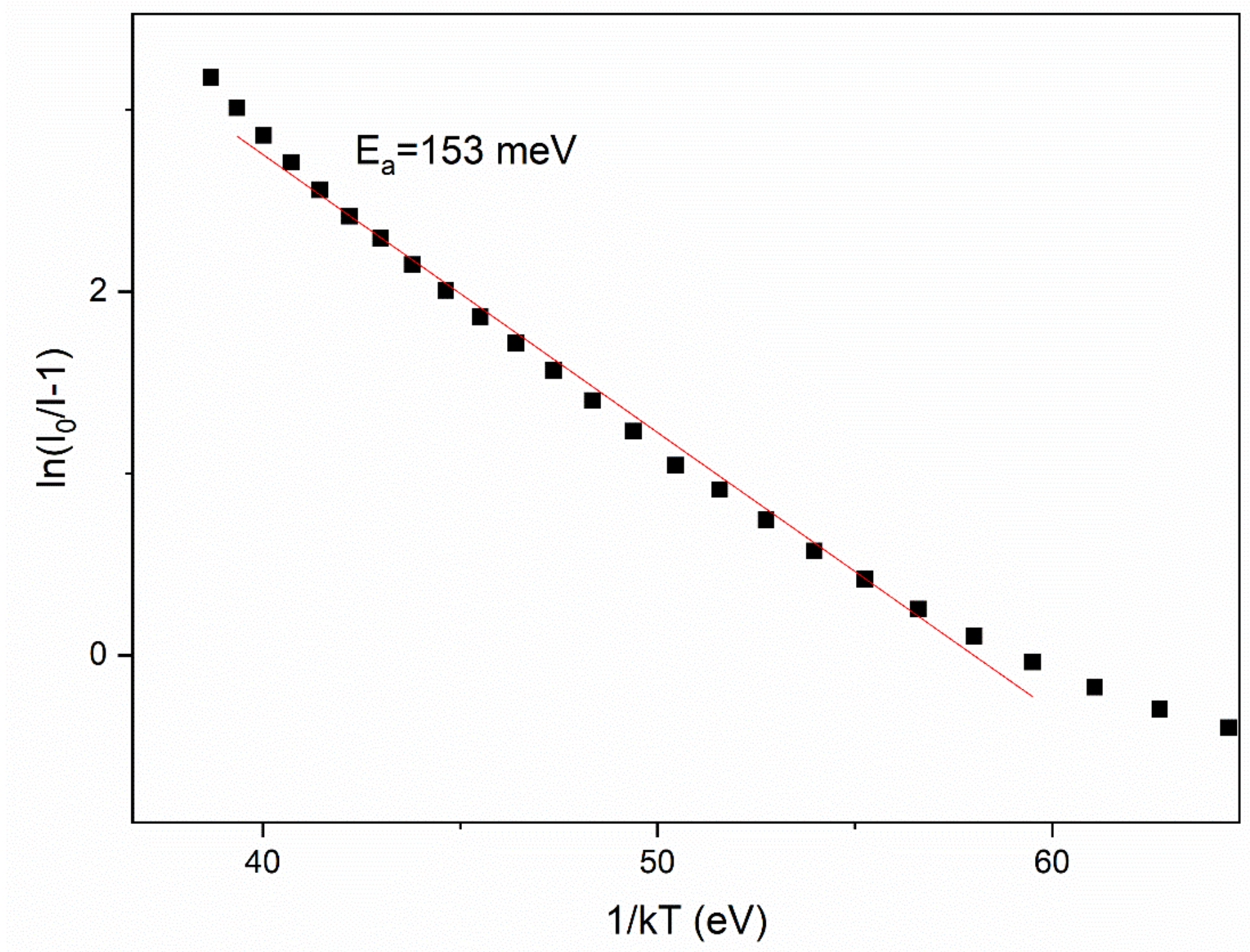

Figure S9. Energy activation for thermal quenching of MPDA-GA analog.

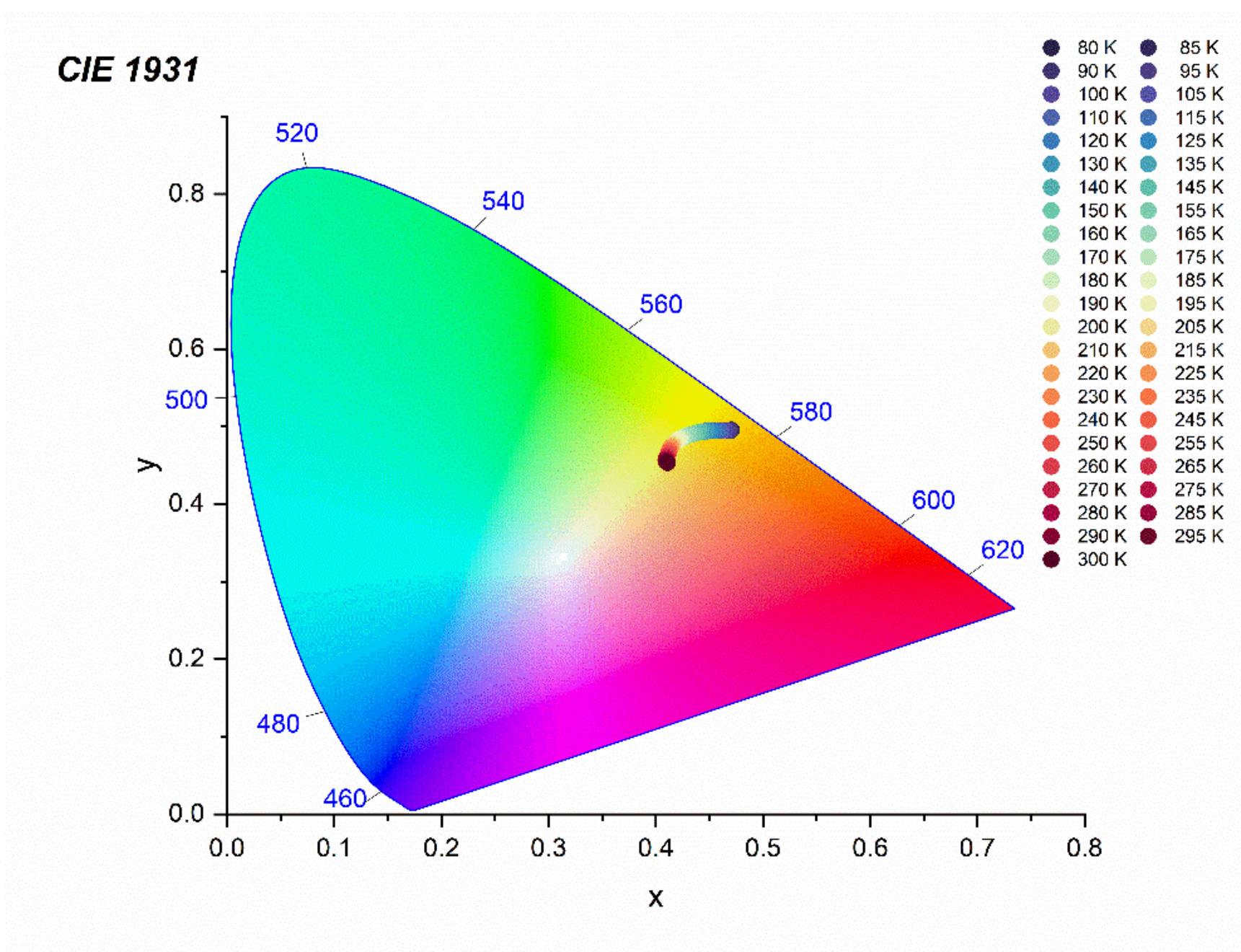

Figure S10. Changes of CIE chromaticity in the function of the temperature of $MPDA_6GA_6Pb_7Br_{30}Br_2$.

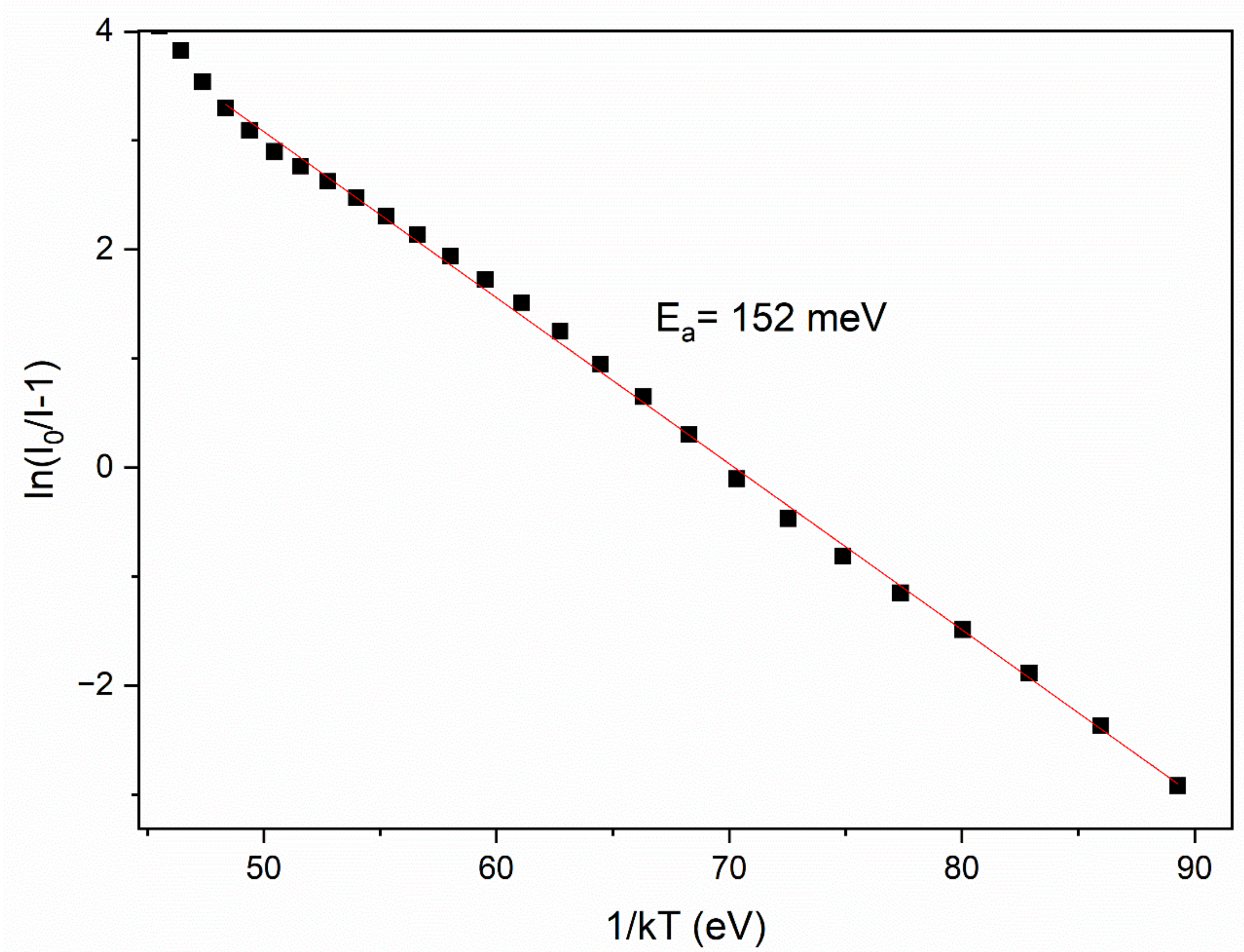

Figure S11. Energy activation for thermal quenching of $MPDA_2IM_3Pb_4Br_{15}$.

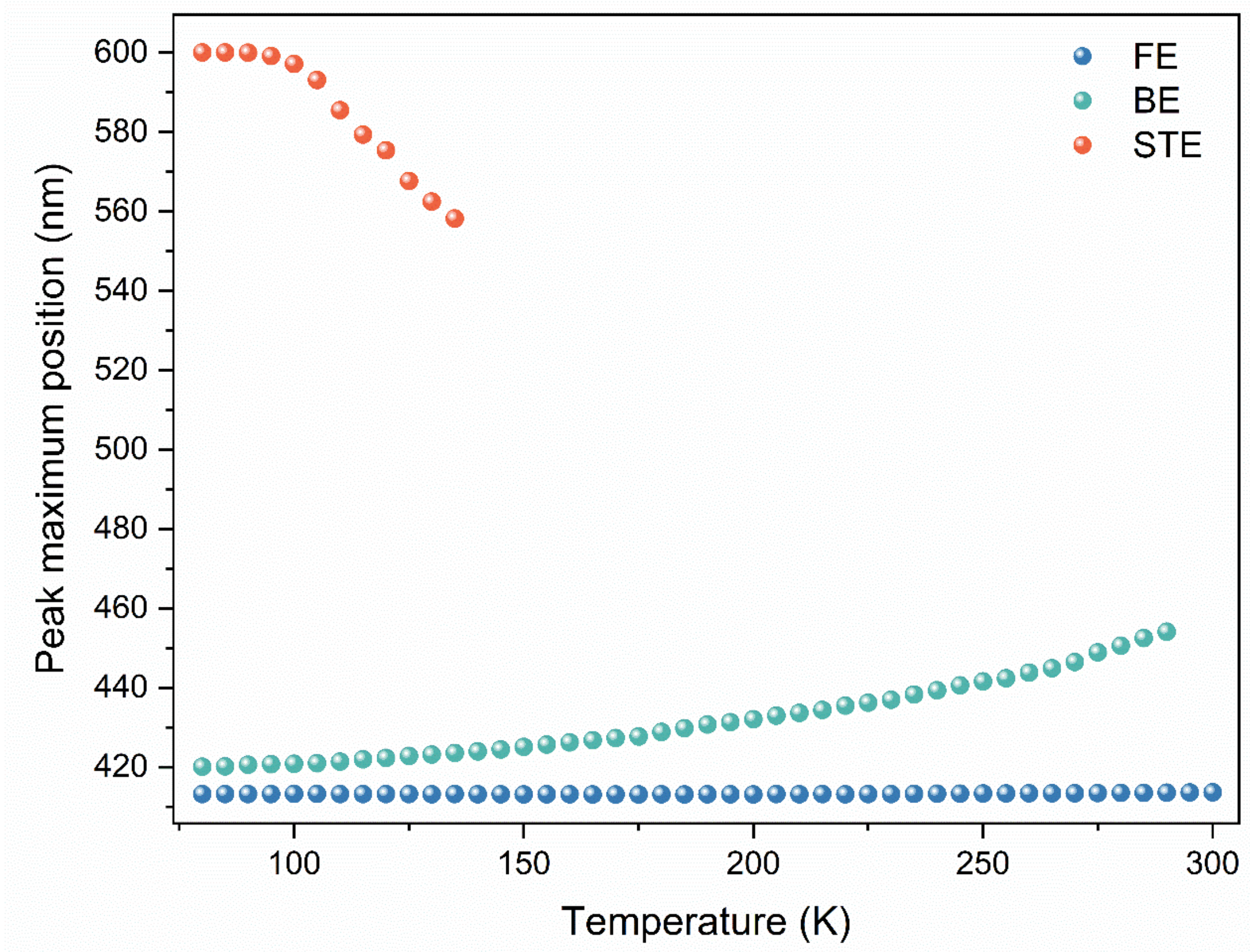

Figure S12. Change in the position of the maximum emission bands of the $MPDA_2IM_3Pb_4Br_{15}$ sample with increasing temperature.

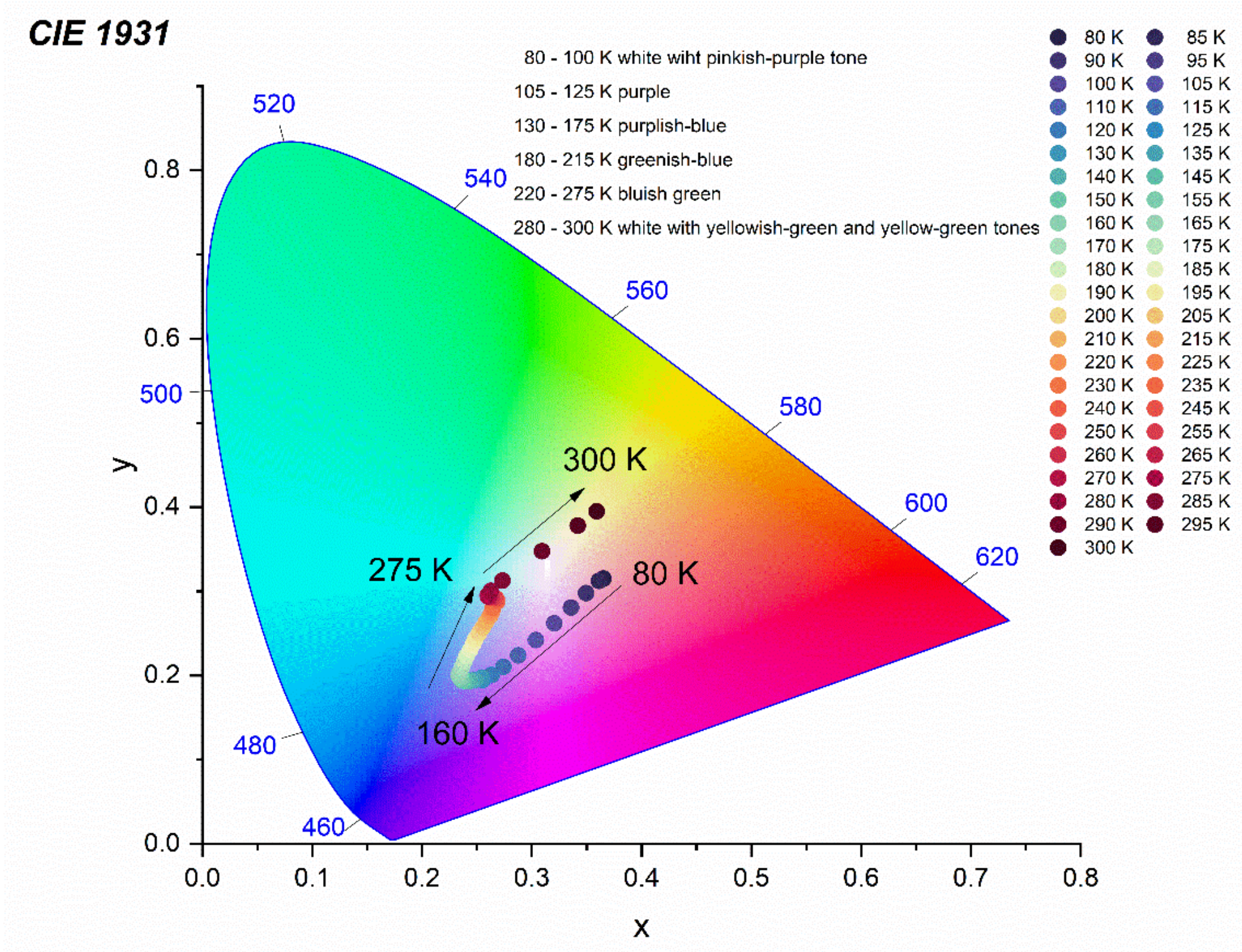

Figure S13. Evolutions of CIE chromaticity in the function of the temperature of $MPDA_2IM_3Pb_4Br_{15}$.

Table S1. Experimental and refinement details of $\mathbf{MPDA_2IM_3Pb_4Br_{15}}$ (M_r = 2414.96 g/mol).

	**Phase II**		**Phase I**
Temperature (K)	100	270	310
**Crystal system, space group**	Monoclinic, $P2_1/n$		Monoclinic, $P2_1/c$
a, b, c (Å)	12.012 (4), 13.936 (4), 29.970 (7)	12.073 (4), 14.064 (4), 30.106 (8)	6.087 (3), 14.353 (4), 29.355 (7)
β (°)	91.66 (3)	91.94 (3)	91.50 (3)
V (Å^3)	5015 (2)	5109 (3)	2563.8 (16)
Z	4		2
μ (mm^{-1})	25.37	24.90	24.81
Crystal size (mm)	0.32 × 0.2 × 0.1		
**Data collection**			
T_{min}, T_{max}	0.017, 0.134	0.034, 0.101	0.041, 0.102
No. of measured, independent and observed [$I > 2\sigma(I)$] reflections	90652, 8846, 6851	27932, 4753, 3239	11458, 2370, 1939
R_{int}	0.076	0.083	0.056
$(\sin \theta/\lambda)_{max}$ (Å^{-1})	0.595	0.481	0.481
**Refinement**			
$R[F^2 > 2\sigma(F^2)]$, $wR(F^2)$, S	0.034, 0.063, 1.02	0.040, 0.085, 1.05	0.037, 0.090, 1.07
No. of reflections	8846	4753	2370
No. of parameters	373	361	177
No. of restraints	12	101	37
$\Delta\rho_{max}$, $\Delta\rho_{min}$ (e Å^{-3})	1.90, -1.87	0.99, -0.92	0.87, -0.97

Table S2. Experimental and refinement details of $\mathbf{MPDA_6GA_6Pb_7Br_{30}Br_2}$ (M_r = 4909.00 g/mol).

**Crystal system, space group**	Trigonal, $P\bar{3}c1$
a, b, c (Å)	17.827 (5), 17.827 (5), 20.652 (6)
α, β, γ (°)	90, 90, 120
V (Å^3)	5684 (4)
Z	2
μ (mm^{-1})	21.62
Crystal size (mm)	0.46 × 0.22 × 0.07
**Data collection**	
T_{min}, T_{max}	0.016, 0.234
No. of measured, independent and observed [$I > 2\sigma(I)$] reflections	125190, 3888, 2963

R_{int}	0.125
(sin $\theta/\lambda)_{max}$ (Å^{-1})	0.625
**Refinement**	
$R[F^2 > 2\sigma(F^2)]$, $wR(F^2)$, S	0.035, 0.057, 1.08
No. of reflections	3888
No. of parameters	165
No. of restraints	50
$\Delta\rho_{max}$, $\Delta\rho_{min}$ (e Å^{-3})	0.69, -0.98

Table S3. Selected geometric parameters of **$MPDA_2IM_3Pb_4Br_{15}$** (Å, º).

Phase II, 100 K		Phase II, 270 K		Phase I, 310 K	
Pb2—Br4^{i}	3.0141 (13)	Pb2—Br4^{i}	3.030 (2)	Pb1—Br2	3.0489 (9)
Pb2—Br6	3.0068 (12)	Pb2—Br6	3.021 (2)	Pb1—Br3^{vi}	3.017 (2)
Pb2—Br9	2.9526 (12)	Pb2—Br9	2.962 (2)	Pb1—Br3	3.076 (2)
Pb2—Br2^{ii}	3.0276 (13)	Pb2—Br2^{ii}	3.033 (2)	Pb1—Br5	3.053 (2)
Pb2—Br7	2.9163 (12)	Pb2—Br7	2.912 (2)	Pb1—Br4	2.985 (2)
Pb2—Br8	3.0759 (12)	Pb2—Br8	3.094 (2)	Pb1—Br1	2.937 (2)
Pb1—Br4	3.0105 (13)	Pb1—Br4	3.025 (2)	Pb2—Br7^{vi}	3.076 (2)
Pb1—Br6	3.0034 (11)	Pb1—Br6	3.021 (2)	Pb2—Br7	3.021 (2)
Pb1—Br2	3.0018 (13)	Pb1—Br2	3.019 (2)	Pb2—Br5	3.249 (2)
Pb1—Br5	2.9422 (12)	Pb1—Br5	2.928 (2)	Pb2—Br4^{i}	3.263 (2)
Pb1—Br3	3.0587 (13)	Pb1—Br3	3.086 (2)	Pb2—Br6	2.800 (2)
Pb1—Br1	2.9635 (12)	Pb1—Br1	2.982 (2)	Pb2—Br8	2.827 (2)
Pb4—Br12	3.0625 (13)	Pb4—Br12	3.061 (2)		
Pb4—Br14	2.8032 (11)	Pb4—Br14	2.806 (2)		
Pb4—Br8^{iii}	3.1475 (12)	Pb4—Br8^{iii}	3.168 (2)		
Pb4—Br15	2.9634 (13)	Pb4—Br15	2.989 (2)		
Pb4—Br13	2.9020 (12)	Pb4—Br13	2.899 (2)		
Pb3—Br12	2.9527 (13)	Pb3—Br12	2.980 (2)		
Pb3—Br9	3.1293 (11)	Pb3—Br9	3.157 (2)		
Pb3—Br10	2.8629 (11)	Pb3—Br10	2.875 (2)		
Pb3—Br15^{iv}	3.0939 (13)	Pb3—Br15^{iv}	3.084 (2)		
Pb3—Br11	2.8485 (11)	Pb3—Br11	2.850 (2)		
Pb3—Br3^{v}	3.2286 (12)	Pb3—Br3^{v}	3.225 (2)		
Br4^{i}—Pb2—Br2^{ii}	168.44 (3)	Br4^{i}—Pb2—Br2^{ii}	169.54 (7)	Br2—Pb1—Br3^{vi}	84.48 (4)
Br4^{i}—Pb2—Br8	88.70 (4)	Br4^{i}—Pb2—Br8	88.38 (7)	Br2—Pb1—Br5	94.33 (4)
Br6—Pb2—Br4^{i}	83.56 (3)	Br6—Pb2—Br4^{i}	84.05 (6)	Br3—Pb1—Br2	94.00 (4)
Br6—Pb2—Br2^{ii}	93.06 (3)	Br6—Pb2—Br2^{ii}	93.04 (6)	Br3—Pb1—Br3^{vi}	175.08 (7)

Br6—Pb2—Br8	93.52 (3)	Br6—Pb2—Br8	94.22 (7)	Br3—Pb1—Br5	97.38 (6)
Br9—Pb2—Br4^{i}	94.76 (3)	Br9—Pb2—Br4^{i}	94.13 (6)	Br5—Pb1—Br3^{vi}	87.41 (6)
Br9—Pb2—Br6	174.82 (3)	Br9—Pb2—Br6	175.16 (7)	Br4—Pb1—Br2	174.85 (5)
Br9—Pb2—Br2^{ii}	87.63 (3)	Br9—Pb2—Br2^{ii}	87.96 (6)	Br4—Pb1—Br3	87.85 (5)
Br9—Pb2—Br8	91.34 (3)	Br9—Pb2—Br8	90.21 (7)	Br4—Pb1—Br3^{vi}	93.27 (5)
Br2^{ii}—Pb2—Br8	102.57 (4)	Br2^{ii}—Pb2—Br8	101.87 (7)	Br4—Pb1—Br5	90.20 (6)
Br7—Pb2—Br4^{i}	83.33 (4)	Br7—Pb2—Br4^{i}	83.70 (7)	Br1—Pb1—Br2	89.31 (5)
Br7—Pb2—Br6	89.04 (3)	Br7—Pb2—Br6	89.78 (7)	Br1—Pb1—Br3	92.50 (6)
Br7—Pb2—Br9	85.89 (3)	Br7—Pb2—Br9	85.56 (7)	Br1—Pb1—Br3^{vi}	82.81 (6)
Br7—Pb2—Br2^{ii}	85.57 (4)	Br7—Pb2—Br2^{ii}	86.25 (7)	Br1—Pb1—Br5	169.20 (6)
Br7—Pb2—Br8	171.31 (3)	Br7—Pb2—Br8	170.72 (7)	Br1—Pb1—Br4	85.81 (7)
Br4—Pb1—Br3	93.81 (4)	Br4—Pb1—Br3	94.16 (7)	Br7—Pb2—Br7^{vi}	173.64 (8)
Br6—Pb1—Br4	96.72 (3)	Br6—Pb1—Br4	95.81 (6)	Br7^{vi}—Pb2—Br5	99.29 (6)
Br6—Pb1—Br3	89.57 (3)	Br6—Pb1—Br3	90.39 (7)	Br7—Pb2—Br5	86.92 (6)
Br2—Pb1—Br4	178.19 (3)	Br2—Pb1—Br4	178.15 (7)	Br7^{vi}—Pb2—Br4^{i}	88.51 (5)
Br2—Pb1—Br6	82.00 (3)	Br2—Pb1—Br6	83.40 (6)	Br7—Pb2—Br4^{i}	89.66 (5)
Br2—Pb1—Br3	87.46 (4)	Br2—Pb1—Br3	87.52 (7)	Br5—Pb2—Br4^{i}	94.06 (6)
Br5—Pb1—Br4	89.52 (4)	Br5—Pb1—Br4	89.71 (7)	Br6—Pb2—Br7	91.66 (6)
Br5—Pb1—Br6	86.11 (3)	Br5—Pb1—Br6	86.34 (7)	Br6—Pb2—Br7^{vi}	89.77 (6)
Br5—Pb1—Br2	89.11 (4)	Br5—Pb1—Br2	88.58 (7)	Br6—Pb2—Br5	89.60 (6)
Br5—Pb1—Br3	174.82 (3)	Br5—Pb1—Br3	175.17 (6)	Br6—Pb2—Br4^{i}	176.26 (7)
Br5—Pb1—Br1	90.95 (4)	Br5—Pb1—Br1	89.09 (7)	Br6—Pb2—Br8	90.66 (8)
Br1—Pb1—Br4	92.63 (3)	Br1—Pb1—Br4	91.34 (6)	Br8—Pb2—Br7	86.55 (6)
Br1—Pb1—Br6	170.17 (3)	Br1—Pb1—Br6	171.49 (7)	Br8—Pb2—Br7^{vi}	87.24 (6)
Br1—Pb1—Br2	88.58 (3)	Br1—Pb1—Br2	89.32 (6)	Br8—Pb2—Br5	173.46 (6)
Br1—Pb1—Br3	92.84 (3)	Br1—Pb1—Br3	93.71 (7)	Br8—Pb2—Br4^{i}	85.62 (8)
Br12—Pb4—Br8^{iii}	97.61 (4)	Br12—Pb4—Br8^{iii}	95.96 (6)	Pb1^{iii}—Br2—Pb1	180.0
Br14—Pb4—Br12	93.04 (3)	Br14—Pb4—Br12	92.69 (7)	Pb1—Br3—Pb1^{iv}	175.08 (8)
Br14—Pb4—Br8^{iii}	87.36 (3)	Br14—Pb4—Br8^{iii}	87.35 (7)	Pb2—Br7—Pb2^{iv}	173.64 (8)
Br14—Pb4—Br15	94.84 (3)	Br14—Pb4—Br15	94.15 (7)	Pb1—Br5—Pb2	169.44 (7)
Br14—Pb4—Br13	85.51 (4)	Br14—Pb4—Br13	86.09 (8)		
Br15—Pb4—Br12	171.33 (3)	Br15—Pb4—Br12	172.64 (7)		
Br15—Pb4—Br8^{iii}	86.38 (4)	Br15—Pb4—Br8^{iii}	87.08 (7)		
Br13—Pb4—Br12	87.91 (4)	Br13—Pb4—Br12	88.32 (7)		
Br13—Pb4—Br8^{iii}	171.21 (3)	Br13—Pb4—Br8^{iii}	172.33 (7)		
Br13—Pb4—Br15	89.10 (4)	Br13—Pb4—Br15	89.44 (7)		
Br12—Pb3—Br9	90.46 (3)	Br12—Pb3—Br9	91.31 (6)		
Br12—Pb3—Br15^{iv}	170.73 (3)	Br12—Pb3—Br15^{iv}	171.51 (7)		
Br12—Pb3—Br3^{v}	85.74 (3)	Br12—Pb3—Br3^{v}	87.03 (6)		
Br9—Pb3—Br3^{v}	87.75 (3)	Br9—Pb3—Br3^{v}	88.95 (6)		

Br10—Pb3—Br12	87.13 (3)	Br10—Pb3—Br12	87.37 (7)		
Br10—Pb3—Br9	82.18 (3)	Br10—Pb3—Br9	81.98 (7)		
Br10—Pb3—Br15^{iv}	84.21 (3)	Br10—Pb3—Br15^{iv}	84.61 (7)		
Br10—Pb3—Br3^{v}	167.61 (3)	Br10—Pb3—Br3^{v}	169.23 (7)		
Br15^{iv}—Pb3—Br9	91.63 (3)	Br15^{iv}—Pb3—Br9	90.34 (6)		
Br15^{iv}—Pb3—Br3^{v}	103.36 (3)	Br15^{iv}—Pb3—Br3^{v}	101.33 (6)		
Br11—Pb3—Br12	86.43 (3)	Br11—Pb3—Br12	87.14 (7)		
Br11—Pb3—Br9	174.43 (3)	Br11—Pb3—Br9	174.32 (7)		
Br11—Pb3—Br10	93.03 (4)	Br11—Pb3—Br10	92.49 (8)		
Br11—Pb3—Br15^{iv}	90.74 (3)	Br11—Pb3—Br15^{iv}	90.41 (7)		
Br11—Pb3—Br3^{v}	96.62 (4)	Br11—Pb3—Br3^{v}	96.42 (7)		
Pb3—Br12—Pb4	171.22 (3)	Pb3—Br12—Pb4	173.49 (9)		
Pb1—Br4—Pb2^{i}	175.86 (3)	Pb1—Br4—Pb2^{i}	176.83 (9)		
Pb1—Br6—Pb2	171.31 (4)	Pb1—Br6—Pb2	173.27 (9)		
Pb2—Br9—Pb3	161.18 (4)	Pb2—Br9—Pb3	162.51 (9)		
Pb1—Br2—Pb2^{ii}	169.22 (4)	Pb1—Br2—Pb2^{ii}	170.21 (9)		
Pb2—Br8—Pb4^{iii}	162.89 (4)	Pb2—Br8—Pb4^{iii}	165.33 (9)		
Pb4—Br15—Pb3^{vi}	168.68 (4)	Pb4—Br15—Pb3^{vi}	170.69 (9)		
Pb1—Br3—Pb3^{vii}	167.28 (4)	Pb1—Br3—Pb3^{vii}	167.25 (9)		

Symmetry codes: (i) -*x*+1, -*y*+1, -*z*+1; (ii) -*x*+2, -*y*+1, -*z*+1; (iii) -*x*+1, -*y*, -*z*+1; (iv) *x*+1, *y*, *z*; (v) *x*, *y*-1, *z*; (vi) *x*-1, *y*, *z*; (vii) *x*, *y*+1, *z*.

Table S4. Selected geometric parameters of $\mathbf{MPDA_6GA_6Pb_7Br_{30}Br_2}$ (Å, º).

Pb2—Br6^{i}	2.9835 (9)	Pb1—Br1	2.9508 (9)
Pb2—Br6^{ii}	2.9836 (9)	Pb1—Br4	3.1403 (7)
Pb2—Br6	2.9836 (9)	Pb1—Br2	2.8355 (9)
Pb2—Br6^{iii}	2.9835 (9)	Pb1—Br5	2.8643 (9)
Pb2—Br6^{iv}	2.9835 (9)	Pb1—Br6	3.1916 (10)
Pb2—Br6^{v}	2.9836 (9)	Pb1—Br3	3.2080 (7)
Br6^{i}—Pb2—Br6^{iii}	93.74 (3)	Br1—Pb1—Br3	87.24 (2)
Br6^{iv}—Pb2—Br6^{ii}	93.74 (3)	Br4—Pb1—Br6	91.50 (2)
Br6—Pb2—Br6^{v}	86.26 (3)	Br4—Pb1—Br3	92.13 (2)
Br6^{i}—Pb2—Br6^{iv}	86.26 (3)	Br2—Pb1—Br1	86.30 (2)
Br6^{i}—Pb2—Br6^{v}	93.74 (3)	Br2—Pb1—Br4	91.03 (2)
Br6^{iii}—Pb2—Br6^{iv}	86.26 (3)	Br2—Pb1—Br5	92.81 (3)
Br6^{iii}—Pb2—Br6^{ii}	180.00 (3)	Br2—Pb1—Br6	177.45 (2)
Br6^{i}—Pb2—Br6	180.0	Br2—Pb1—Br3	93.37 (3)
Br6—Pb2—Br6^{ii}	93.74 (3)	Br5—Pb1—Br1	89.47 (3)
Br6^{iii}—Pb2—Br6	86.26 (3)	Br5—Pb1—Br4	91.46 (3)

Br6^{iv}—Pb2—Br6^{v}	180.0	Br5—Pb1—Br6	87.43 (3)
Br6^{iv}—Pb2—Br6	93.74 (3)	Br5—Pb1—Br3	172.79 (2)
Br6^{ii}—Pb2—Br6^{v}	86.26 (3)	Br6—Pb1—Br3	86.23 (3)
Br6^{i}—Pb2—Br6^{ii}	86.26 (3)	Pb1^{vi}—Br4—Pb1	162.02 (4)
Br6^{iii}—Pb2—Br6^{v}	93.74 (3)	Pb2—Br6—Pb1	168.47 (3)
Br1—Pb1—Br4	177.217 (17)	Pb1—Br3—Pb1^{vii}	165.38 (4)
Br1—Pb1—Br6	91.17 (3)		

Symmetry codes: (i) -*x*+2, -*y*+2, -*z*+1; (ii) -*x*+*y*+1, -*x*+2, *z*; (iii) *x*-*y*+1, *x*, -*z*+1; (iv) -*y*+2, *x*-*y*+1, *z*; (v) *y*, -*x*+*y*+1, -*z*+1; (vi) *y*, *x*, -*z*+1/2; (vii) -*x*+2, -*x*+*y*+1, -*z*+1/2.

Table S5. Selected hydrogen bonding parameters of **$MPDA_2IM_3Pb_4Br_{15}$** (Å, º).

D—H···*A*	*D*—H (Å)	H···*A* (Å)	*D*···*A* (Å)	*D*—H···*A* (°)
**Phase II, 100 K**				
N32—H32A···Br10^{i}	0.91	2.60	3.441 (7)	153.6
N32—H32B···Br15^{ii}	0.91	2.62	3.419 (7)	147.5
N32—H32C···Br2^{iii}	0.91	2.54	3.425 (8)	165.0
N28—H28A···Br12	0.91	2.90	3.447 (8)	120.3
N28—H28A···Br11	0.91	2.63	3.411 (8)	144.7
N28—H28B···Br13	0.91	2.64	3.471 (7)	152.5
N21—H21D···Br4^{iv}	0.91	2.57	3.455 (8)	163.1
N21—H21E···Br12	0.91	2.54	3.391 (7)	155.6
N21—H21F···Br10	0.91	2.73	3.530 (8)	147.6
N21—H21C···Br7	0.91	2.59	3.461 (7)	160.7
N19—H19···Br7^{iv}	0.88	2.74	3.426 (8)	136.1
N16—H16···Br8	0.88	2.49	3.360 (7)	171.7
N13—H13···Br5^{v}	0.88	2.47	3.272 (8)	152.5
N11—H11···Br3	0.88	2.50	3.324 (8)	156.0
N25—H25A···Br13^{vi}	0.91	2.74	3.458 (10)	136.8
N25—H25B···Br5^{iv}	0.91	2.44	3.314 (12)	160.3
**Phase II, 270 K**				
N32—H32A···Br10^{i}	0.89	2.62	3.505 (14)	175.1
N32—H32B···Br15^{ii}	0.89	2.90	3.455 (16)	122.3
N32—H32C···Br2^{iii}	0.89	2.75	3.471 (15)	139.3
N28—H28A···Br12	0.89	2.87	3.427 (16)	121.7
N28—H28A···Br11	0.89	2.78	3.543 (17)	144.0
N28—H28B···Br13	0.89	2.68	3.495 (16)	153.5
N21—H21E···Br12	0.89	2.71	3.447 (15)	141.0
N21—H21F···Br7	0.89	2.66	3.543 (15)	171.1
N21—H21B···Br10	0.89	2.91	3.596 (14)	135.4

N21—H21C···Br4^{iv}	0.89	2.71	3.481 (15)	145.7
N19—H19···Br7^{iv}	0.86	2.83	3.51 (2)	137.4
N16—H16···Br8	0.86	2.57	3.414 (18)	167.6
N13—H13···Br5^{v}	0.86	2.51	3.297 (18)	153.2
N11—H11···Br3	0.86	2.59	3.405 (19)	157.8
N25A—H25A···Br5^{iv}	0.89	2.43	3.28 (3)	162.3
N25A—H25B···Br13^{vi}	0.89	2.73	3.44 (3)	137.6
N25—H25C···Br15^{vi}	0.89	2.93	3.59 (8)	132.6
**Phase I, 310 K**				
N6—H6···Br1	0.86	2.67	3.384 (17)	141.0
N8—H8···Br5^{vii}	0.86	2.56	3.409 (16)	169.3

Symmetry codes: (i) -*x*+3/2, *y*-1/2, -*z*+3/2; (ii) -*x*+1/2, *y*-1/2, -*z*+3/2; (iii) *x*-1/2, -*y*+1/2, *z*+1/2; (iv) -*x*+1, -*y*+1, -*z*+1; (v) -*x*+2, -*y*+1, -*z*+1; (vi) -*x*+1/2, *y*+1/2, -*z*+3/2; (vii) -*x*, -*y*, -*z*+1.

Table S6. Selected hydrogen bonding parameters of **$MPDA_6GA_6Pb_7Br_{30}Br_2$** (Å, º).

D*—H···*A	***D*—H (Å)**	**H···*A* (Å)**	***D*···*A* (Å)**	***D*—H···*A* (°)**
N8—H8A···Br2^{i}	0.86	2.81	3.607 (2)	155.6
N8—H8B···Br2	0.86	2.81	3.607 (2)	155.6
N11—H11A···Br5^{ii}	0.86	2.63	3.486 (6)	170.7
N9—H9A···Br2	0.86	2.85	3.642 (7)	153.5
N9—H9B···Br4^{ii}	0.86	2.82	3.625 (6)	155.7
N13—H13A···Br3^{iii}	0.86	2.75	3.555 (2)	156.2
N13—H13B···Br3	0.86	2.75	3.555 (2)	156.2
N5—H5A···Br7^{iv}	0.89	2.43	3.244 (6)	151.4
N5—H5B···Br1^{v}	0.89	2.53	3.414 (6)	173.4
N1—H1AA···Br6^{iii}	0.89	2.69	3.434 (7)	141.8
N1—H1AC···Br1^{iii}	0.89	2.48	3.358 (7)	170.7
N1—H1BE···Br6^{v}	0.89	2.65	3.502 (8)	160.9
C4—H4A···Br2^{vi}	0.97	2.90	3.712 (9)	142.3
C2—H2A···Br3^{iii}	0.97	2.91	3.75 (2)	146.0

Symmetry codes: (i) -*x*+2, -*x*+*y*+1, -*z*+1/2; (ii) -*x*+*y*+1, -*x*+1, *z*; (iii) -*x*+*y*+1, -*x*+2, *z*; (iv) -*x*+1, -*y*+1, -*z*+1; (v) *y*, -*x*+*y*+1, -*z*+1; (vi) *y*, *x*, -*z*+1/2.
